# At the “Peak” of Vis-to-UV Upconversion: Clear Advantages of TIPS Substituents for a Biphenyl Annihilator

**DOI:** 10.1021/jacsau.5c01202

**Published:** 2025-11-07

**Authors:** Julian A. Moghtader, Masanori Uji, Till J. B. Zähringer, Matthias Schmitz, Luca M. Carrella, Alexander Heckel, Eva Rentschler, Nobuhiro Yanai, Christoph Kerzig

**Affiliations:** † Department of Chemistry, 9182 Johannes Gutenberg University Mainz, Duesbergweg 10−14, 55128 Mainz, Germany; ‡ Department of Chemistry, Graduate School of Science, 13143 The University of Tokyo, 7-3-1 Hongo, Bunkyo-ku, Tokyo 113-0033, Japan; § Institute for Organic Chemistry and Chemical Biology, 9173 Goethe University Frankfurt, Max-von-Laue-Str. 7, 60438 Frankfurt am Main, Germany

**Keywords:** Energy Transfer, Fluorescence Spectroscopy, Kinetics, Photochemistry, Time-Resolved Spectroscopy

## Abstract

Sensitized triplet–triplet annihilation systems enable the efficient conversion of two low-energy photons from a low-intensity, noncoherent light source into one high-energy photon, opening avenues for diverse applications. The attachment of triisopropylsilylethynyl (TIPS-ethynyl) groups to aromatic compounds has led to the development of many annihilators with high upconversion quantum yields. Here, we synthesized a series of novel symmetrical biphenyls bearing silylethynyl substituents of varying sizes and evaluated them as annihilators in visible-to-UV upconversion systems. While all of these systems do not suffer from excimer formation issues, their upconversion performances differ significantly. Small substituents like trimethylsilylethynyl give similar upconversion quantum yields (up to ∼12%) as the TIPS-ethynyl groups, but the larger triphenylsilylethynyl substituents reduce the achievable quantum yields by half, which is likely due to additional nonradiative loss channels arising from altered energies of higher excited triplet states. The UV emission of the novel annihilators is hypsochromically shifted by about 20 nm relative to that of the TIPS-naphthalene benchmark UV annihilator, thereby reaching a highly attractive spectral region for bond activation photochemistry. Using the most efficient annihilator, bTIPS-BP, we achieved blue-to-UV upconversion-driven release of fluorescein from a photocage. In the greater context of photon upconversion and in view of other recent reports on substituent effects, our results indicate that several chromophore-specific effects must be understood for obtaining optimized systems.

## Graphical abstract

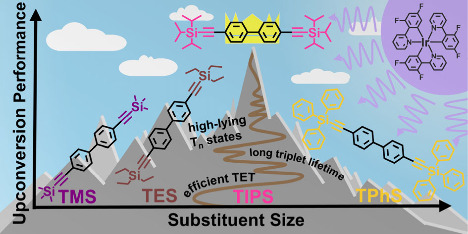

## Introduction

Photon upconversion is a highly potent and versatile concept that can improve the performance of many contemporary technologies, such as photovoltaics, bioimaging, 3D printing, and various bond activation processes for chemical applications.^1–12^ Many different strategies like two-photon absorption (2PA), lanthanide-based upconversion nanoparticles (UCNPs), nonlinear optics, or sensitized triplet–triplet annihilation upconversion (sTTA-UC) can be utilized for the absorption of two low-energy photons and their conversion into one high-energy photon.^13–17^ Among these approaches, sTTA-UC shows great application potential since upconversion can operate under low incident excitation intensities utilizing noncoherent light sources.^18–21^ Owing to the need for high-energy photons in a multitude of applications, blue-to-UV upconversion is an especially relevant topic.^17,22–26^ The first blue-to-UV upconversion system via sensitized triplet–triplet annihilation upconversion was originally explored by Castellano et al. in 2006.^27^ A system consisting of Ir­(ppy)_3_ as sensitizer (Sens) and 1,6-di-*tert*-butylpyrene as triplet energy acceptor and annihilator (An) was used, which laid the grounds for the implementation of blue-to-UV sTTA in chemical applications that would otherwise rely on high-energy UV excitation.^18,28–30^ The interest in such upconversion systems received a further boost by the discovery of TIPS-naphthalene as a benchmark annihilator by Kimizuka and Yanai, which, in combination with the sensitizer Ir­(C6)_2_(acac), yielded the first highly efficient blue-to-UV upconversion system with achievable quantum yields ϕ_UC_ beyond 10%.^31^ With numerous recent application examples of blue-to-UV upconversion systems, such as in 3D printing, wastewater treatment, photocatalysis, and biomedical applications,^26,32–34^ the interest in the development of novel, even more efficient vis-to-UV upconversion systems is still increasing.^35–41^ Especially blue-to-UV upconversion systems generating wavelengths as short as possible are desired, where the high energy emission can photoexcite and activate a broad range of compounds and can overcome energy barriers that are inaccessible for visible-light driven systems.^42–44^ A few upconversion systems that utilize sTTA-UC to generate photons in the UV-B or even the UV-C region have been developed (see Figure 1), but they lag behind in upconversion efficiency, leading to an enormous efficiency falloff for annihilators with emission wavelengths <365 nm. UV upconversion systems face challenges owing to the intrinsic nature of UV chemistry, such as quick degradation as well as increased filter effects. Moreover, the high excited-state energies seem to be responsible for inherent loss channels during the TTA process that still have to be understood. However, this spectral range is highly attractive because commercial high-power LEDs with peak emission wavelengths <365 nm are not available.^45–49^ This work extends the range of efficient upconversion (ϕ_UC_ > 10% in the linear region of the intensity dependence) systems to ∼350 nm in the UV region (emission onset at ∼325 nm, compare Figure 1), while simultaneously further exploring the influence of different substituents at the silylethynyl groups on potential annihilators.^50,51^

**Figure 1:**
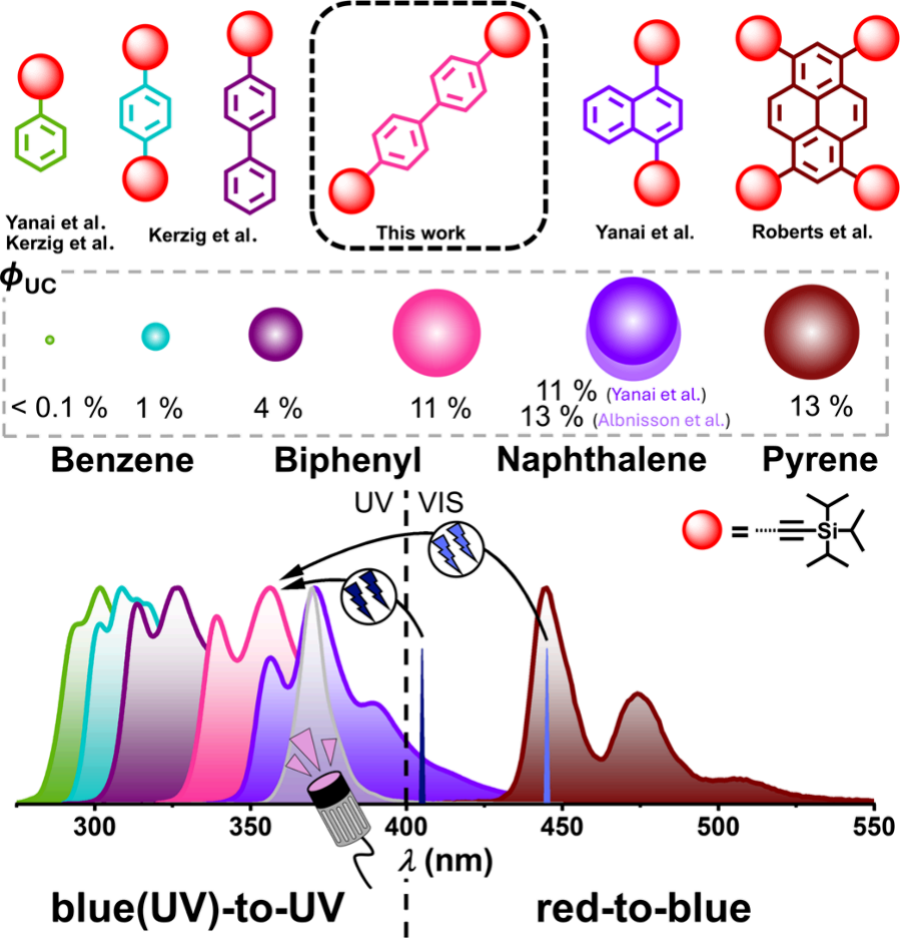
Emission spectra and external upconversion quantum yields ϕ_UC_ of TIPS-ethynyl (triisopropylsilylethynyl)-disubstituted biphenyl in comparison to already published annihilators used in upconversion systems that are based on TIPS-ethynyl modification (ϕ_UC_ is not corrected for filter effects; at infinite excitation intensities with a theoretical limit of 50%).^31,45,46,49,51–53^ Vertical lines indicate the excitation wavelengths of the cw lasers used in this study. The gray spectrum was recorded for a 370 nm high-power LED; commercial high-power LEDs with significantly shorter emission wavelengths are not available. See the text for further explanations.

Recently, many studies have focused on the development of novel triplet annihilation chromophores, which revealed that the modification of an organic chromophore with **TIPS**-ethynyl (triisopropylsilylethynyl) groups is a viable pathway to synthesize novel annihilators with outstanding upconversion characteristics.^45,46,49,52,54–57^ However, the exact role with all its advantages and disadvantages of this group is to this day only partially understood.^50,58–60^ Owing to their high electron density, triisopropylsilylethynyl groups increase the singlet–triplet gap while expanding the amount of π-electrons minimally. This in turn increases the maximum achievable anti-Stokes shift for an upconversion system.^57,61–66^ Additionally, the silyl substitution leads to improved fluorescence quantum yields ϕ_Fl_ for chromophores, thereby increasing the upconversion quantum efficiency of the respective upconversion system accordingly.^67,68^ Albinsson et al. systematically substituted naphthalene at the 1,4 positions with different substituents at the silylethynyl moiety to explore the steric influence of methyl, isopropyl, and phenyl groups.^50^ They concluded that bulkier substituents hamper excimer formation, leading to improved upconversion efficiencies. Interestingly, **TIPS**- and triphenylsilyl (**TPhS**)-ended ethynyl groups had essentially identical effects on the performance of the investigated mono- and disubstituted naphthalene annihilators. For trialkylsilylethynyl derivatives of pyrene, Roberts and co-workers showed that the smaller trimethylsilylethynyl (**TMS**-ethynyl) substituent leads to the most efficient upconversion, despite competitive excimer formation occurring.^51^ While several structural modifications were tested to fine-tune bimolecular rate constants and the excited-state energies or to avoid excimer formation^69–71^ for the frequently studied perylene annihilator chromophore in the past, the positive influence of **TIPS**-ethynyl groups was investigated only very recently.^50,51,57^ On the other hand, recent results from Pun and co-workers suggest that some annihilator chromophores benefit from rather flat substituent “straps”, which reduce the core-to-core distance and increase the spin-statistical *f* value of an annihilator.^72,73^ Additional factors that could have an impact on the *f* value such as molecular orientation during the TTA process and minor effects on the excited-state energy landscape have barely been explored.^74–76^

As we will show, the effect of the different substituents at the silylethynyl groups is completely different for the novel biphenyl annihilator compared to what has been observed for pyrene and naphthalene derivatives. Specifically, the annihilator with two **TIPS**-ethynyl groups shows the most promising annihilator properties and outperforms the corresponding **TPhS** compound, which is explained by the different energies of higher-lying triplet states promoting loss channels in the **TPhS** derivative. We additionally exploit the excellent performance of the most efficient annihilator for a blue-light-driven uncaging reaction of **fluorescein**, which would otherwise require UV light.

## Results and Discussion

As the organic annihilator chromophore, we have chosen biphenyl and doubly substituted the core structure with four different silylethynyl groups, namely trimethylsilylethynyl (**TMS**), triethylsilylethynyl (**TES**), triisopropylsilylethynyl (**TIPS**), and triphenylsilylethynyl (**TPhS**) to yield the respective bis­(trialkyl/phenyl)­silylethynyl-biphenyl (**bTXS-BP**, Figure 2A). The novel annihilator compounds were prepared by Sonogashira coupling reactions starting from 4,4′-dibromobiphenyl, and their identities and purities were analyzed by mass spectrometry and ^1^H as well as ^13^C NMR spectroscopy. All analytical data sets are shown in the Supporting Information (SI, section 4). For **bTPhS-BP**, a crystal structure could be obtained after vapor diffusion crystallization (SI, section 5).

Trialkylsilyl groups are generally known to enhance fluorescence quantum yields.^67^ When compared to unsubstituted biphenyl and mono-TIPS-substituted biphenyl (TIPS-biphenyl), this holds true: Biphenyl emits 15% of its initially absorbed photons, while TIPS-biphenyl has a fluorescence quantum yield (ϕ_Fl_) of 48% in nonpolar solvents.^52,77^ Accordingly, all four **bTXS-BP** compounds show high fluorescence quantum yields of ∼90% in strongly diluted cyclohexane and toluene solutions with a short singlet state lifetime τ_Fl_ of ∼0.7 ns (Table 1).

**Table 1:** Photophysical Properties of the Disubstituted Biphenyls

	bTMS-BP	bTES-BP	bTIPS-BP	bTPhS-BP
S_1_ (eV)^a^,^b^	3.79	3.78	3.78	3.75
τ_Fl_ (ns)^a^,^c^	0.71	0.69	0.67	0.64
Φ_Fl, 50 μM_ (%)^a^,^d^	90.1	90.5	90.8	90.1
T_1_ (eV)^e^	2.31 (2.40)	– (2.46)	– (2.40)	2.31 (2.40)
T_1_ → T_*n*(Abs,max)_ (nm)^a^,^f^	451	457	461	478

a Measured in toluene.

b The excited singlet state energy is estimated by the intersection of the normalized absorption and emission spectra.

c Measured using TCSPC, with a 293 nm EPLED as excitation source.

d Determined by the absolute fluorescence quantum yield method using a concentration of 50 μM, see SI, Section 1.6.

e Determined at the high-energy emission peak of the phosphorescence spectra measured in 2-methyl-tetrahydrofuran. Based on these measurements and the calculated triplet energy levels shown in parentheses, essentially identical triplet energies are expected for all derivatives.

f Transient absorption maximum of the respective T_1_ state using laser flash photolysis.

When the four newly synthesized chromophores are compared to each other, only minor differences can be found. Not only is this true for the fluorescence properties and the energies of the lowest excited singlet state (S_1_), but DFT calculations also predict that all four chromophores show similar properties with respect to their triplet states, as indicated by spin density and triplet energy calculations (SI, section 1.12). This was confirmed by 77 K measurements (see SI, Figure S13), which revealed a triplet energy of 2.31 eV for both **bTMS-BP** and **bTPhS-BP**, which is very similar to the calculated triplet energies (Table 1). UV–vis absorption spectroscopy showed that all chromophores have similar absorption bands with only **bTPhS-BP** being slightly red-shifted (Figure 2B). This makes all four compounds equally attractive to test them as annihilators for sTTA-UC.

**Figure 2:**
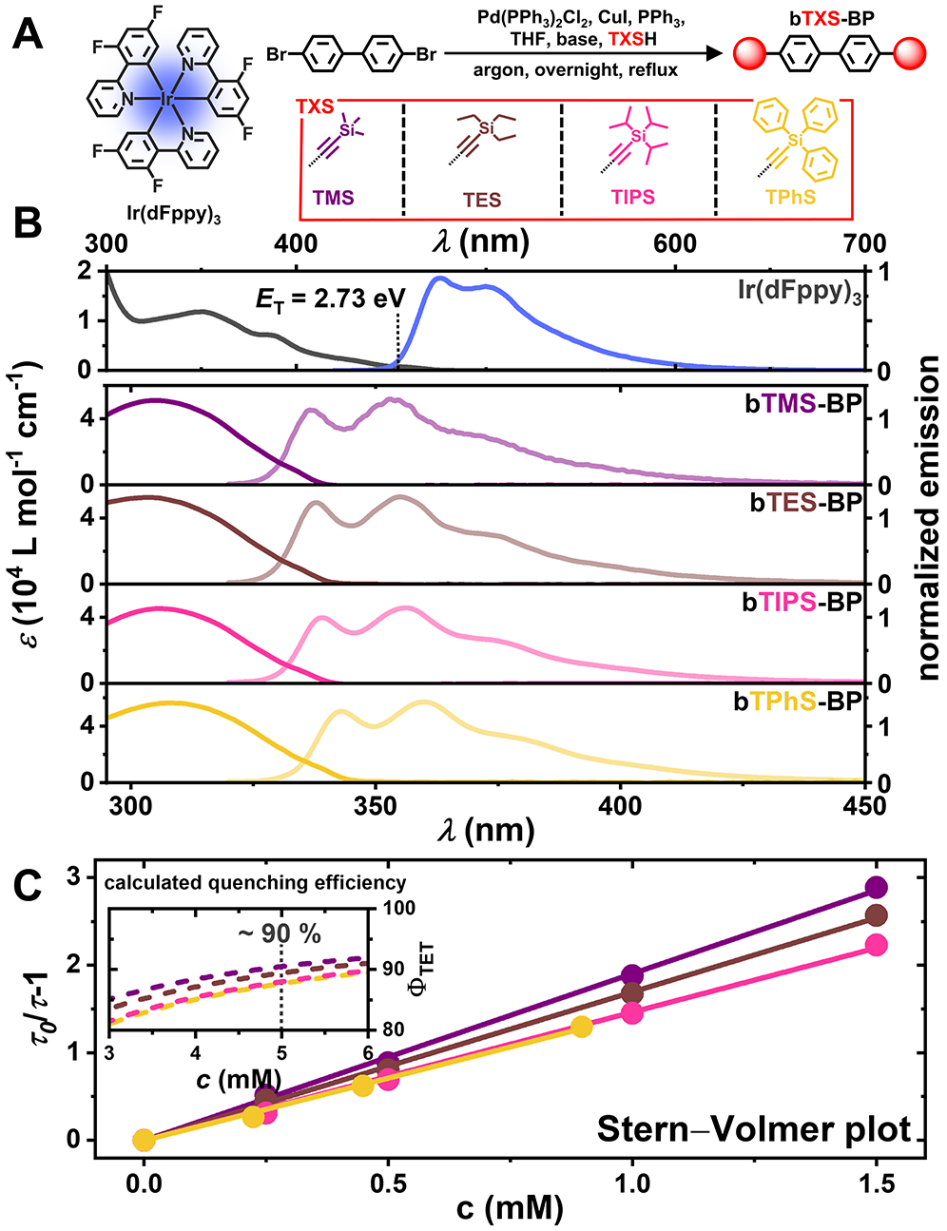
(A) Molecular structure of tris­[3,5-difluoro-2-(2-pyridinyl)­phenyl]-iridium (**Ir­(dFppy)**_**3**_) and different biphenyl substituents **TXS** with the reaction scheme for the synthesis of the annihilators. (B) UV–vis absorption and normalized emission spectra of the sensitizer **Ir­(dFppy)**_**3**_ and the annihilators (top to bottom): 4,4′-bis­((trimethylsilyl)­ethynyl)­biphenyl (**bTMS-BP**, purple), 4,4′-bis­((triethylsilyl)­ethynyl)­biphenyl (**bTES-BP**, brown), 4,4′-bis­((triisopropylsilyl)­ethynyl)­biphenyl (**bTIPS-BP**, pink), and 4,4′-bis­((triphenylsilyl)­ethynyl)­biphenyl (**bTPhS-BP**, yellow). (C) Stern–Volmer plot of ^**3**^ **Ir­(dFppy)**_**3**_ quenching with different annihilator concentrations in toluene. The expected relative errors for the resulting quenching rate constants are below 5%. The inset shows the calculated quenching efficiency as a function of the annihilator concentration.

Based on the above-mentioned annihilator properties, we have selected **Ir­(dFppy)**_**3**_ as a sensitizer for our upconversion measurements. This complex still absorbs in the blue region of the visible spectrum while retaining a high triplet energy of 2.73 eV in toluene (Figure 2B), which would lay the grounds for a fast triplet energy transfer (TET) from **Ir­(dFppy)**_**3**_ to the respective biphenyl derivative. Additionally, an efficient back-triplet energy transfer cannot proceed since ∼0.4 eV would be needed to undergo this endergonic process, whereas room temperature can only enable uphill energy transfer with energy differences of up to about 0.3 eV.^78–84^ Choosing **Ir­(dFppy)**_**3**_ might not be ideal for application-related usage, since reabsorption effects and a low molar absorption coefficient at the incident excitation wavelength (see Figure S19) reduce the achievable absolute upconversion quantum yield (ϕ_UC_) and increase the upconversion threshold intensity (*I*_th_). However, for comparative studies focusing on the inherent annihilator properties, our sensitizer choice is expected to be well-suited owing to its above-mentioned characteristics and high photostability. Stern–Volmer analyses were employed for all sensitizer–annihilator pairs, substantiating that the triplet energies of all four derivatives are close to each other. We observed merely a slight difference between the smallest biphenyl **bTMS-BP** and the largest quencher **bTPhS-BP**. While **bTMS-BP** quenches ^**3**^ **Ir­(dFppy)**_**3**_ with a rate constant *k*_q_ = 1.35 × 10^9^ M^–1^ s^–1^, the quenching rate constant of **bTPhS-BP** was determined as *k*_q_ = 1.01 × 10^9^ M^–1^ s^–1^ (Figure 2C). The difference in quenching rates can most likely be explained by the increased size and molecular weight that are introduced by the bulkier substituents: Compared to **bTPhS-BP**, **bTMS-BP** is significantly smaller (see also the comparative DFT-calculated structures in Figure S2), which leads to a higher diffusion rate through the solvent and therefore an improved rate for diffusion-based quenching. Steric hindrance and therefore limited accessibility of energy donor and acceptor orbitals are seemingly minor issues. At lower quencher concentrations, this discrepancy in the quenching rate can lead to similarly large differences in the quenching efficiency, but when an adequate quencher concentration is chosen, the difference shrinks to marginal values. For our selected standard conditions with 5 mM of the respective biphenyl derivative as quencher, this translates to a varying quenching efficiency between 90.5% (for **bTMS-BP**) and 87.7% (for **bTPhS-BP**, see Figure 2C, inset). This minor difference in the overall quenching efficiency should only influence the upconversion studies marginally.

We then turned to laser flash photolysis (LFP) measurements for analyzing the quenching mechanism of the reaction between ^**3**^ **Ir­(dFppy)**_**3**_ and **bTXS-BP** and for identifying the quenching products. For this, we first compared the TA spectrum of ^**3**^ **Ir­(dFppy)**_**3**_ after selective 355 nm excitation with transient absorption spectra in the presence of 3 mM **bTES-BP** as a representative quencher (Figure 3A). On the time scale of 1 μs, the transient absorption spectrum shifts from transient absorption signals that can be assigned to the triplet state of **Ir­(dFppy)**_**3**_ with an apparent absorption maximum at 447 nm (due to emission contaminations in the TA spectrum), to the triplet state of **bTES-BP** with an absorption maximum at 457 nm.^45,52^ A snapshot of the spectral emission after 0.85 μs additionally reveals that emission from both **Ir­(dFppy)**_**3**_ and **bTES-BP** can be detected (Figure 3B). For **Ir­(dFppy)**_**3**_, this emission stems from the well-known room-temperature phosphorescence (which is not quenched completely in the given detection window). **bTES-BP** most likely exhibits delayed fluorescence resulting from sTTA-UC, given that the excited state singlet lifetime amounts to only ∼0.7 ns for which no emission should be visible in that detection time window even if direct excitation would play a role. These results were confirmed by kinetic transient absorption and emission measurements at representative detection wavelengths (Figure 3C). Here, the phosphorescence emission of **Ir­(dFppy)**_**3**_ decays with the same rate as the formation of ^**3**^ **bTES-BP**. The emission from ^**1**^ bTES-BP* rises and decays with a nonlinear dependence on the ^**3**^ **bTES-BP** concentration, a behavior that is typical for sTTA-UC processes.^85^ While basic LFP experiments were performed with all biphenyl derivatives, more sophisticated concentration-dependent transient absorption measurements were carried out with **bTMS-BP** as the quencher and annihilator to find out whether excimer formation of the triplet state with a secondary molecule in the ground state can occur. Even at 20-times increased annihilator concentrations, no changes in the triplet state lifetime and the TA spectrum of the T_1_ state could be detected during these measurements with standardized initial triplet concentrations (see SI, Figures S20 and S21).^50^ Furthermore, the fluorescence quantum yield is only very slightly reduced when the **bTMS-BP** concentration increased from 50 μM to 5 mM from 90.1% to 87.7%. This minor change rules out excimer formation between a singlet-excited and a ground-state annihilator molecule and can be traced back to filter effects. Based on the absence of excimer formation for the sterically least hindered annihilator, we assume that excimer formation does not play any role for all annihilators of our study.^50,58^ The transient absorption spectra of the biphenyl derivatives measured following TET show identical band structure, but their absorption maxima as well as their relative absorption coefficients vary (Figure 3D). On a photophysical basis, these observed triplet absorption bands can be assigned to T_1_ → T_*n*_ transitions. The intensities of the absorption bands correlate with the electronic transition moments and the overlap integrals of the wave functions for nuclear vibrations of the T_1_ states with their respective T_*n*_ states.^86^ With an increasing substituent size, these transient absorption bands undergo a bathochromic shift from 451 nm for **bTMS-BP** to 478 nm for **bTPhS-BP**. Considering our aforementioned findings, this seems to be the only pronounced energetic difference between these different biphenyl derivatives.

**Figure 3:**
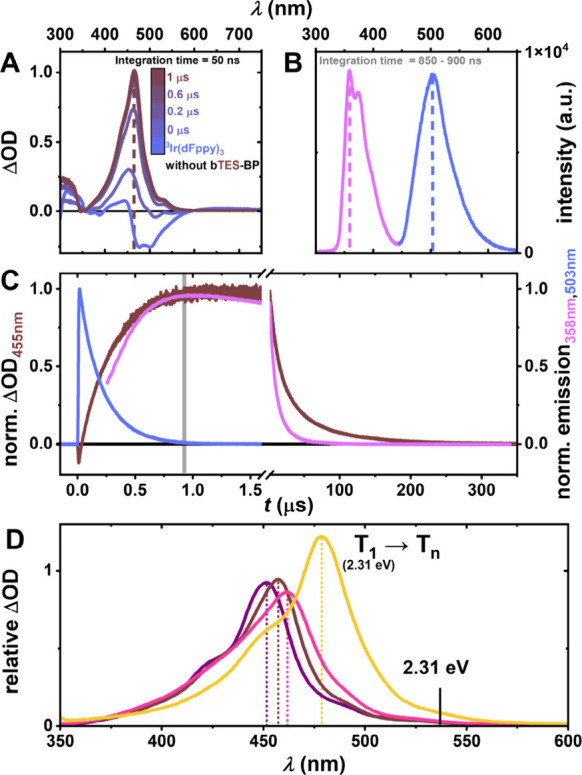
(A) Transient absorption (TA) spectra of a deaerated solution of 25 μM **Ir­(dFppy)**_**3**_ and 3 mM **bTES-BP** in toluene at different delay times after laser excitation with 355 nm pulses (16 mJ, ∼5 ns). For comparison, a TA spectrum of only 25 μM **Ir­(dFppy)**_**3**_ in deaerated toluene is shown (light blue). (B) An emission spectrum of the same solution with a delay of 0.85 μs and time-integration over 50 ns is shown (detection window highlighted in panel C). (C) Normalized kinetic transient absorption (brown) and emission (blue, pink) decay traces of the same solution are shown. (D) TA spectra of 5 mM **bTMS-BP** (purple), **bTES-BP** (brown), **bTIPS-BP** (pink), and **bTPhS-BP** (yellow) in toluene with 30 μM **Ir­(dFppy)**_**3**_ as sensitizer in deaerated toluene. The spectra were recorded 1 μs after excitation (100 ns integration), where emission of **Ir­(dFppy)**_**3**_ was absent, indicating completion of the TET process. All other parameters such as laser intensity, detector settings, and probe lamp intensity were kept constant to yield spectra whose intensities reflect relative molar absorption coefficients.

For quantitative upconversion studies, the samples were prepared in an argon-filled glovebox to ensure identical conditions. To further substantiate the high (>85%) and very similar energy transfer quantum yields Φ_TET_ that we calculated with the Stern–Volmer equation (see above), measurements of the absolute quantum yield of the remaining (quenched) sensitizer emission were carried out. The resulting Φ_TET_ values (see Table 2) are very similar compared to the theoretical predictions shown in Figure 2C. In Figure 4A, the external (measured) upconversion quantum yield is plotted against the laser intensity (for raw data sets and selected upconversion spectra, see section 3.5 of the SI). Here, **bTIPS-BP** shows the highest experimentally measured upconversion quantum yield (ϕ_UC,ext_) of ∼9.9%, with measured upconversion quantum yields of ∼7.8%, ∼7.3%, and ∼2.8% for **bTMS-BP**, **bTES-BP**, and **bTPhS-BP**, respectively (with a maximum upconversion quantum yield of 50%).^87^ In Figure 4B, the upconversion emission is plotted against the laser intensity to reveal the threshold intensities *I*_th_ of the systems under study. Since only **bTIPS-BP** reaches a slope of 1 at high excitation intensities which would permit a simplified analysis, the *I*_th_ values of all samples were approximated using the fitting function developed by Murakami and Kamada.^85^ This procedure revealed a threshold intensity of 2.7 W cm^–2^ for the system employing **bTIPS-BP**, while the other systems had threshold intensities exceeding 10 W cm^–2^ (see Table 2). In principle, a discrepancy in *I*_th_ values can be explained by the differences in triplet lifetimes *and* varying triplet–triplet annihilation rate constants of the individual triplet annihilators (see Figure S25 and related text for further explanations). The triplet lifetimes can be extracted from the upconversion emission lifetime at low excitation light intensities (Figure 4C). With an increased triplet lifetime, the threshold intensity is reduced (quadratically).^88^ This behavior is qualitatively in good agreement with our measurements (Figure 4C), since **bTIPS-BP** shows the longest triplet excited state lifetime.

**Figure 4:**
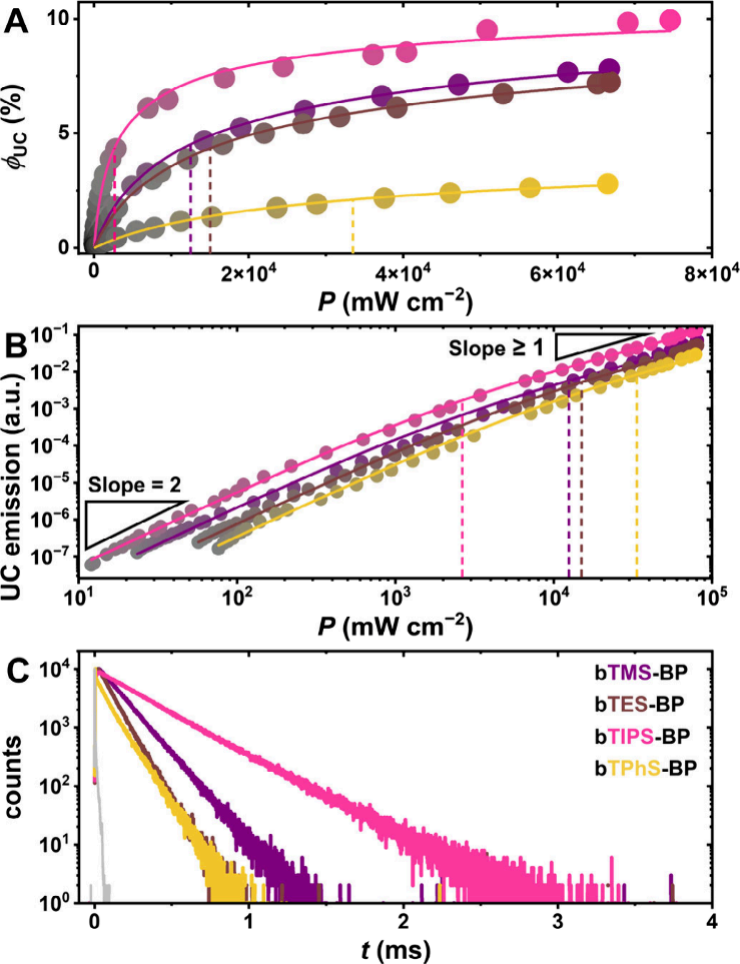
Triplet annihilation upconversion studies of deaerated solutions containing 50 μM **Ir­(dFppy)**_**3**_ and 5 mM **bTXS-BP** in toluene using a 405 nm cw laser as an excitation source. (A) External quantum efficiency ϕ_UC, ext_ plotted against the excitation intensity. (B) Upconversion emission intensity plotted against the excitation intensity in a double logarithmic fashion. (C) Emission lifetime measurements of the upconversion emission, excited with a xenon flash lamp and equipped with a 445 nm band-pass filter and neutral density filters in the excitation beam. The sharp initial decrease in the lifetime curve for **bTPhS-BP** is a stray light artifact and does not affect the triplet lifetime determination.

**Table 2:** Key Parameters of Upconversion Systems Consisting of 50 μM Ir­(dFppy)_3_ and 5 mM bTXS-BP in Toluene upon 405 nm cw Laser Excitation

	bTMS-BP	bTES-BP	bTIPS-BP	bTPhS-BP
Φ_UC,ext_ (%)	7.8	7.3	9.9	2.8
Φ_UC,∞_ (%)^a^	11.8	11.4	11.4	5.5
*I*_th_ (W cm^–2^)^a^	12.5	15.0	2.7	33.5
Φ_Fl,An_ (%)	87.7	84.8	90.0	77.2
Φ_Em,Sens_ (%)	5.5	8.0	7.2	6.7
Φ_TET_ (%)^b^	92.8	89.6	90.6	91.3
*f* (%)^c^	20.2	21.5	20.4	11.7
T_1_ → T_*n*(Abs,2.31eV)_ ^d^	1.0	1.0	1.3	3.6
τ_An_ (ms)^e^	0.67	0.45	1.47	0.49

a Estimated with experimental data as described by Murakami and Kamada.^85^

b Determined by the residual emission Φ_Em,Sens_ of **Ir­(dFppy)**_**3**_ in the sample.

c This value represents the calculated lower limit of the *f* value, since it is only corrected for the reabsorption of the annihilator. For further details, see text and SI, Section 3.5.

d Relative molar absorption coefficient of the T_1_ → T_*n*_ transition at the energy of the T_1_ state determined from the transient absorption spectra displayed in Figure 3 D; normalized to 1. See text for further explanations.

e Determined utilizing TCSPC, see SI Section 1.8.

Since our upconversion measurement setup (see SI, section 1.5) did not allow us to increase the excitation intensity for all of our upconversion systems to reach their linear regime, we extrapolated our upconversion data to give us a theoretical external upconversion quantum yield maximum at an infinite excitation intensity (ϕ_UC,∞_).^85^ Here, the three compounds **bTMS-BP**, **bTES-BP**, and **bTIPS-BP** show nearly the same upconversion quantum yields at infinite excitation intensity ϕ_UC,∞_ of 11.8%, 11.4%, and 11.4%, respectively. Only **bTPhS-BP** differs extensively with ϕ_UC,∞_ = 5.5% (see Table 2). Utilizing these data sets, we estimated the spin statistical factor *f*. This was done with the following formula: ϕ_UC,∞_ = 1/2 × *f* × ϕ_ISC_ × ϕ_TET_ × ϕ_TTA_ × ϕ_Fl_.^72^ Here, ϕ_ISC_ and ϕ_TTA_ are assumed to be unity, since **Ir­(dFppy)**_**3**_ is known for a near-quantitative intersystem crossing quantum yield and an infinitely high excitation intensity leads to ϕ_TTA_ values close to unity as photophysical triplet deactivation cannot compete anymore.^89–91^ We stress that the resulting *f* values have to be regarded as lower limits as we did not correct the upconversion quantum yields for optical losses mainly resulting from the reabsorption of the upconverted photons by the sensitizer (which strongly absorbs in the near UV region), from FRET, and from the usage of a 400 nm short-pass filter in our experimental setup (see Table S3 and related text for further explanations). However, we stress that these losses do not affect the comparability of the relative *f* values within our annihilator series. For **bTMS-BP**, **bTES-BP**, and **bTIPS-BP**, values of *f* = 20.2, 21.5, and 20.4% were found, respectively, while **bTPhS-BP** yielded *f* = 11.7%. An explanation for this discrepancy is provided by utilizing the differences in the observed T_1_ → T_*n*_ absorption properties. A competitive process to the triplet annihilation 2 × T_1_ → S_1_ is the nonradiative recombination to a higher triplet state 2 × T_1_ → T_*n*_. This process is especially competitive when the energy difference from 2 × T_1_ to T_*n*_ is small.^59,92^ When comparing the transient absorption spectra (i.e., the absorption spectra of T_1_) of the biphenyl derivatives, one can see that the triplet absorption spectrum of **bTPhS-BP** at the energy level of the excited triplet state (T_1_ = 2.31 eV = 537 nm; corresponding to the transition to a T_*n*_ state at 4.62 eV) is more than twice as intense compared to all other compounds (see Figure 3 D), indicating a stronger transition moment for T_1_ → T_*n*_ at that energy level. This directly translates into a more competitive 2 × T_1_ → T_*n*_ loss pathway within the annihilation event and might explain the discrepancy in the spin statistical factor *f* between **bTPhS-BP** and all of the other biphenyl derivatives. The crystal structure that we obtained for **bTPhS-BP** revealed that two annihilator molecules can indeed accommodate a structure with the π-systems of two adjacent annihilator molecules in close proximity. The 7.4 Å distance between two aromatic core units (Figure S39, SI) should still enable productive annihilation considering that typical distances for the triplet pair on the order of 3–7 Å were reported for other annihilators.^41,72,88,93^ Based on preliminary measurements, the triplet–triplet annihilation rate *k*_TTA_ seems to be slightly lower for **bTPhS-BP** compared to **bTIPS-BP** and much lower than that of **bTMS-BP**, either owing to the increased steric hindrance of the phenyl groups or as a result of slower diffusion (see SI, section 3.8). A more reliable analysis of all rate constants is difficult to achieve with our equipment. However, we believe that a recently developed technique with modulated cw diode lasers providing square-shaped pulses and simultaneous time-resolved emission detection is highly promising for the simultaneous determination of *k*_TTA_ and many other upconversion parameters in follow-up studies.^94^ The reduced *k*_TTA_ value for **bTPhS-BP**, in conjunction with the even more drastically reduced triplet lifetime, negatively impacts the threshold intensity *I*_th_ and reduces application potential with low-intensity excitation sources. Based on our measurements, we conclude that **bTIPS-BP** shows the most beneficial properties as an annihilator for sTTA-UC. Since all biphenyl derivates suppress excimer formation in the singlet excited state and in the ground state, their achievable upconversion quantum yields can likely be ascribed to their excited state energy landscape. There, the **bTPhS-BP** annihilator suffers from lower lying triplet excited states T_*n*_, which are more easily accessible as a competitive process to productive excited singlet generation from TTA, which inherently limits the upconversion efficiency of this compound to unexpectedly low levels when compared to the other biphenyl-based compounds.

Blue-to-UV upconversion allows the usage of lower energy emission wavelengths for applications that rely on higher energy excitation.^26^ We demonstrate this by employing our most efficient annihilator (**bTIPS-BP**) with the broadly applied sensitizer (**4CzIPN**) to efficiently drive the uncaging reaction of **fluorescein** from its bis­(5-carboxymethoxy-2-nitrobenzyl)­ether fluorescein (**CMNB-caged fluorescein**).^95^ Compared to **Ir­(dFppy)**_**3**_, **4CzIPN** absorbs more strongly in the blue spectral region and reabsorption of the upconverted emission is less pronounced (see Figure S19).^52^ These beneficial properties should enable a lower threshold intensity, while facilitating applications with longer optical path lengths. The **CMNB-caged fluorescein** does not absorb light in the visible region. Upon the absorption of UV photons it cleaves off both (5-carboxymethoxy-2-nitrobenzyl) groups to yield the highly efficient green emitter **fluorescein** in solution.^96,97^ The **fluorescein** release initiated by blue-to-UV upconversion can be detected by emission spectroscopy when exciting the sample in the blue/green region (490 nm in our case). When we directly irradiated a solution of 15 μM **CMNB-caged fluorescein** using a 447 nm cw laser at 1 W output power, almost no emission from the uncaged **fluorescein** could be detected, even after 30 min of irradiation. In contrast, by simply irradiating a degassed cuvette with our upconversion system, consisting of 100 μM **4CzIPN** and 7 mM **bTIPS-BP** (with the same 447 nm light source), next to the cuvette with **CMNB-caged fluorescein**, we were able to induce efficient release of **fluorescein** from its cage (Figure 5B,C). Here, the UC system is employed as light source for a radiative energy transfer (see ref 26 for the mechanistic diversity of UC-driven applications). Further control experiments with only degassed **4CzIPN** as a 447 nm illuminated system led to no conversion (compare Figure S12). Our blue-light-driven UC system thus serves as an effective UV light source: The upconverted emission following the sTTA process between **4CzIPN** and **bTIPS-BP** is reabsorbed by **CMNB-caged fluorescein**, which undergoes bond cleavage upon excitation (Figure 5A). Our blue light-driven system used for this application consisting of **4CzIPN** and **bTIPS-BP** has a high experimentally measured upconversion quantum yield ϕ_UC,ext_ of up to 10.5%, and the threshold intensity is relatively low with a value of 319 mW cm^–2^ (Figure 5D). Considering that the intersystem crossing quantum yield is reduced to ∼70% with **4CzIPN** in the solvent under study (toluene),^52^ the lower limit of *f* for **bTIPS-BP** must be higher than that obtained with the iridium sensitizer (which gave a similar upconversion quantum yield despite an ISC quantum yield approaching unity), which is most likely due to reduced reabsorption effects.^52^ Additionally, the longer-lived triplet state of **4CzIPN** leads to highly efficient triplet energy transfer (see SI, Figure S9), which further enhances the triplet energy transfer efficiency ϕ_TET_. While the purely organic sensitizer clearly has some advantages over the Ir complex, its reduced photostability is a problem for long-term applications. Nevertheless, upconverted emission can still be detected after 30 min even with the highest excitation intensity of the most powerful cw laser used in this study (see SI, section 3.6). The absorption spectrum of **CMNB-caged fluorescein** overlaps in large parts with the upconverted emission spectrum of **bTIPS-BP**, which leads to a high percentage of photons that can be reabsorbed. We increased the concentration of **4CzIPN** in comparison to the upconversion quantum yield measurements to improve the absorption of 447 nm photons, while slightly reducing the upconversion efficiency.^98^ These factors allow efficient uncaging despite a rather simple and nonoptimized setup with two cuvettes next to each other, in which about 92.5% of the upconverted photons do not hit the sample containing **CMNB-caged fluorescein** (see SI for details).^99^

**Figure 5:**
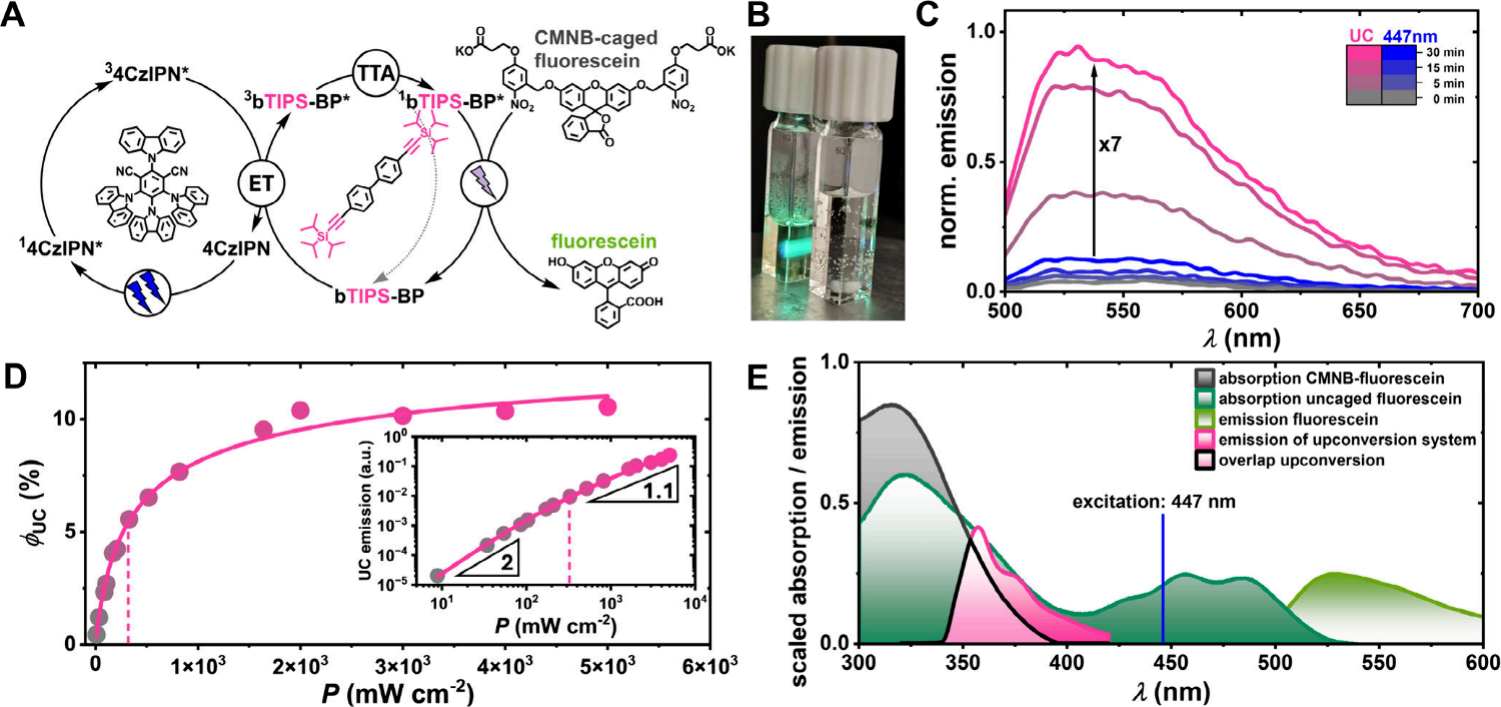
(A) Schematic representation of an sTTA-UC system (**4CzIPN** as sensitizer and **bTIPS-BP** as annihilator) used as a UV light source for the blue light-driven activation of **CMNB-caged fluorescein**. (B) Photograph of the uncaging setup with the upconversion solution consisting of 100 μM **4CzIPN** and 7 mM **bTIPS-BP** in argon-saturated toluene in the left cuvette excited with a blue (447 nm) cw laser and 15 μM **CMNB-caged fluorescein** in H_2_O/DMF (1:1) in the right cuvette. (C) Emission from the cuvette with the uncaging reaction upon excitation at 490 nm after direct irradiation with a 447 nm cw laser (1 W, blue) or indirect irradiation as shown in panel B (pink). (D) External upconversion quantum yield plotted against the incident laser intensity. Inset: Double logarithmic plot of the upconversion emission versus the excitation power density. Measured with the setup described in ref 52. (E) Absorption spectra of **CMNB-caged fluorescein** (black), uncaged **fluorescein** (dark green) and emission spectra of **bTIPS-BP** in the upconversion system (pink) and of uncaged **fluorescein** (light green). The spectral overlap of the UC emission and the photocage absorption is highlighted with black borders.

## Conclusions

In summary, we established a novel biphenyl-based annihilator for visible-to-UV upconversion and investigated the influence of differently sized substituents at the silylethynyl groups on the upconversion performance of the chromophore. We were able to confirm nearly identical photophysical properties for all four compounds, with the only major deviations found in the unquenched triplet lifetimes and the triplet–triplet absorption spectra, indicating that energetically lower T_1_ → T_*n*_ transitions for the sterically larger phenyl substituents enable additional loss channels during the triplet annihilation event. With our novel molecular design, we were able to blue-shift the upconverted emission by about 20 nm compared to the TIPS-naphthalene benchmark compound,^31^ thereby reaching a highly attractive spectral region in which commercial high-power UV-LEDs do not operate anymore. Simultaneously, a high experimentally measured upconversion quantum yield exceeding 10% could be maintained. Our novel annihilator molecule **bTIPS-BP** possesses the most attractive properties for blue-to-UV upconversion, and we exploited these properties through the combination with **4CzIPN** as sensitizer to activate **CMNB-caged fluorescein** in a simple emission-reabsorption setup. With this, we not only demonstrate the beneficial effects of the **TIPS**-group on the properties of an annihilator but also give additional insights into possible limitations of differently substituted silylethynyl groups. In contrast to recent studies with annihilators based on naphthalene and pyrene, excimer issues do not play any role, even with small methyl groups. What is more, long unquenched triplet lifetimes and the energy landscape of higher triplet states are responsible for the unique performance of the novel **bTIPS-BP** annihilator. Our studies also highlight the challenges associated with the identification of an ideal sensitizer for a novel UV annihilator as several factors such as filter effects and photostability must be considered. We hope that our work will contribute to the development of novel and even more efficient upconversion systems for future applications.

## Supplementary Material



## Data Availability

All experimental data have been provided in the main text and the SI. The data sets shown in the main paper and DFT output files can be found under https://doi.org/10.25358/openscience-13600.

## Notes

Experimental details, analytical data, additional steady-state and time-resolved spectroscopic results, single crystal X-ray data and details about the irradiation experiment (PDF)
