# Supplementary material for: At the “Peak” of Vis-to-UV Upconversion: Clear Advantages of TIPS Substituents for a Biphenyl Annihilator
Source: JACS Au. 2025 Nov 7;5(11):5707–16. doi: 10.1021/jacsau.5c01202 (PMC12648284; doi:10.1021/jacsau.5c01202)
Supplement: Supplementary file 1 [file au5c01202_si_001.pdf]

Supporting information

# At the "Peak" of Vis-to-UV Upconversion: Clear Advantages of TIPS Substituents for a Biphenyl Annihilator

Julian A. Moghtader[1], Masanori Uji[2], Till J. B. Zähringer[1], Matthias Schmitz[1], Luca M. Carrella[1], Alexander Heckel[3], Eva Rentschler[1], Nobuhiro Yanai[2*] and Christoph Kerzig[1*]

[1]Department of Chemistry, Johannes Gutenberg University Mainz, Duesbergweg 10 – 14, 55128 Mainz, Germany; Email: ckerzig@uni-mainz.de

[2]Department of Chemistry, Graduate School of Science, The University of Tokyo, 7-3-1 Hongo, Bunkyo-ku, Tokyo 113-0033, Japan; Email: yanai@chem.s.u-tokyo.ac.jp

[3]Institute for Organic Chemistry and Chemical Biology, Goethe University Frankfurt, Max-von-Laue-Str. 7, 60438 Frankfurt am Main, Germany

# Contents


1. General experimental details and methods ........ 1
1.1 Materials ........ 1
1.2 Chromatography ........ 1
1.3 NMR spectroscopy ........ 1
1.4 Steady-state UV-vis and emission measurements ........ 2
1.5 Steady-state sTTA-UC measurements ........ 2
1.6 Fluorescence quantum yield measurements ........ 3
1.7 Low-intensity time-resolved upconversion emission measurements ........ 4
1.8 Time-correlated single photon counting (TCSPC) measurements ........ 4
1.9 Phosphorescence measurements ........ 4
1.10 Irradiation experiments ........ 4
1.11 Laser flash photolysis (LFP) ........ 5
1.12 Theoretical calculations ........ 5
1.13 Field-desorption mass spectrometry ........ 7
2. Synthetic procedure ........ 7
2.1 Synthesis of bTMS-BP ........ 8
2.2 Synthesis of bTES-BP ........ 8
2.3 Synthesis of bTIPS-BP ........ 8
2.4 Synthesis of bTPhS-BP ........ 9
3. Additional spectroscopic data ........ 9
3.1 Stern–Volmer measurements ........ 9
3.2 Upconversion and CMNB-caged fluorescein activation with 4CzIPN and bTIPS-BP ........ 13
3.3 Additional photophysical properties of the annihilators ........ 16
3.4 Upconversion with 445 nm as excitation source ........ 17
3.5 Upconversion emission correction (405 nm excitation) ........ 20
3.6 Stability measurements ........ 22
3.7 Excimer formation ........ 24
3.8 Triplet Annihilation Rate of bTMS-BP, bTIPS-BP and bTPhS-BP ........ 26
4. NMR and mass spectra ........ 28
5. X-ray crystallography ........ 33
6. References ........ 36

# 1. General experimental details and methods

## 1.1 Materials

Unless specifically mentioned, all chemicals employed in synthesis, irradiation experiments or optical spectroscopy were commercially obtained and used as received: chloroform, 99.5 %, titolchimica; tetrahydrofuran, 99.7 %, VWR; toluene, 99.5 %, TCI; toluene, 99.5 %, VWR; cyclohexane, 99.7 %, VWR; N,N-dimethylformamide, 99.9 %, chemsolute; thermo scientific; *p*-terphenyl, 99 %, Acros Organics; 4,4'-dibromobiphenyl, 99 %, Acros Organics; trimethylsilylacetylene, 97 %, Alfa Aesar; triethylsilylacetylene, 97 %, Alfa Aesar; triisopropylsilylacetylene, 97 %, Alfa Aesar; triphenylsilylacetylene, 97 %, Sigma-Aldrich; bistriphenylphosphine- palladium(II)dichloride, 98 %, Acros Organics; copper(I)iodide, 99.9 %, Alfa Aesar; triphenylphosphine, 99 %, Sigma-Aldrich; diisopropylamine , 99.9 %, VWR; triethylamine, 95 %, VWR; Ir(dFppy)$_3$, 95%, Sigma-Aldrich; CMNB-caged carboxyfluorescein, 70 %, Invitrogen; sodium hydrogen carbonate, 99.7 %, fisher scientific; sodium sulfate, 99 %, thermo scientific.

Water was desalinated and then purified using a Millipore Simplicity UV Water Purification System by Merck (21.5 MΩ cm at 20°C).

For NMR measurements the following deuterated solvent was used: chloroform-$d_1$ 99.8%, Deutero.

Argon from Nippon Gases (5.0) was used for removing oxygen from solutions by purging when specifically mentioned.

4CzIPN was previously synthesized and purified in our group and used as is.[1]

## 1.2 Chromatography

For standard liquid column chromatography separation, silica gel 60 M (0.040-0.063 mm Macherey-Nagel GmbH & Co., Düren, Germany) was used. Thin layer chromatography (TLC) was performed using "DC Kieselgel 60 F254" (Merck KGaA, Darmstadt, Germany) on aluminum and a UV lamp (Benda, NU-4 KL, λ = 254 nm and 365 nm, Wiesloch, Germany) was employed for detection.

## 1.3 NMR spectroscopy

For NMR spectroscopy a multinuclear magnetic resonance spectrometer of the type AV II 400 (Bruker, Karlsruhe, Germany) was employed. The chemical shifts are given as δ-values in ppm and they were referenced relative to the residue signal of the non-deuterated solvent.[2,3] The following abbreviations were used: singlet (s), doublet (d), quintet (q) and multiplet (m).

## 1.4 Steady-state UV-vis and emission measurements

UV-vis absorption spectra were recorded on a JASCO V-780 or a JASCO V-750-ST spectrophotometer. Photoluminescence spectra were measured by using a JASCO FP-8300 spectrofluorometer, a Perkin Elmer FL-6500 spectrophotometer or an Edinburgh Instruments FS5 spectrofluorometer.

## 1.5 Steady-state sTTA-UC measurements

For obtaining TTA-UC emission spectra, a diode laser (405 and 445 nm, RGB Photonics) was used as the excitation source. The laser power was controlled by combining a software (Ltune) and a variable neutral density filter and measured using a PD300-UV photodiode sensor (OPHIR Photonics). The laser beam was focused on a sample in a 1 mm cuvette using a lens. The diameters of the laser beam ($1/e^2$) were measured at the sample position using a CCD beam profiler SP620 (OPHIR Photonics). The emitted light was focused by an achromatic lens to an optical fiber connected to a multichannel detector MCPD-9800 (Otsuka Electronics). 425 or 400 nm short-pass filter was used between the sample and the detector. TTA-UC emission spectra above 300 nm were calibrated by using a standard lamp Ocean Optics HL-3 plus-CAL.

The TTA-UC quantum yield ($\phi_{\mathrm{UC}}$) in deaerated toluene was determined by a relative method using a standard, according to the following equation,[4,5]

$$\phi_{\mathrm{UC}} = \Phi_{\mathrm{std}} \left( \frac{1 - 10^{-A_{\mathrm{std}}}}{1 - 10^{-A_{\mathrm{UC}}}} \right) \left( \frac{E_{\mathrm{UC}}}{E_{\mathrm{std}}} \right) \left( \frac{I_{\mathrm{std}}}{I_{\mathrm{UC}}} \right) \left( \frac{n_{\mathrm{UC}}}{n_{\mathrm{std}}} \right)^2$$

where $\Phi$, $A$, $E$, $I$ and $n$ represent quantum yield, absorbance at excitation wavelength, integrated photoluminescence spectral profile, excitation intensity, and refractive index of the solvent, respectively.[4,5] Note that the theoretical maximum of the $\phi_{\mathrm{UC}}$ is 0.5 (50%), *i.e.* the raw data sets were not multiplied by two. The subscripts UC and Std denote the system of upconversion and standard. Sensitizer phosphorescence (Phos) was used as an internal standard, and the efficiency was calculated by the following equation,

$$\phi_{\mathrm{UC}} = \Phi_{\mathrm{Phos}} \left( \frac{E_{\mathrm{UC}}}{E_{\mathrm{Phos}}} \right) \left( \frac{I_{\mathrm{Phos}}}{I_{\mathrm{UC}}} \right)$$

The maximum theoretical upconversion quantum yield $\Phi_{\mathrm{UC},\infty}$, as well as the upconversion threshold intensity $I_{\mathrm{th}}$ was determined by applying the following fit equation by Murakami and Kamada.[6]

$$\phi_{\mathrm{UC}} = \phi_{\mathrm{UC},\infty} \left( 1 + \frac{1 - \sqrt{1 + 4\left(\frac{I_{\mathrm{ex}}}{I_{\mathrm{th}}}\right)}}{2\left(\frac{I_{\mathrm{ex}}}{I_{\mathrm{th}}}\right)} \right)$$

For upconversion measurements utilizing 4CzIPN as sensitizer, a 447 nm 1 W cw laser from Roithner Laser Technik GmbH was used as excitation source for the sample in a 1 cm

cuvette.[1] The laser was positioned at a 90° angle to the Perkin Elmer FL-6500 spectrophotometer as detector. Optical density filters from Thorlabs were used to reduce the emission intensity if needed. The emission of the upconverted sample was compared to a sample only containing 4CzIPN to determine the upconversion quantum yield.

## 1.6 Fluorescence quantum yield measurements

The absolute emission quantum yield was measured in an integrating sphere using a HAMAMATSU multichannel analyzer C10027-01.

Relative fluorescence quantum yield were measured in cyclohexane with *p*-terphenyl as emission standard (reference quantum yield of 93 % in cyclohexane).[7] Cyclohexane as solvent was chosen here to circumvent solvent excitation. The resulting fluorescence quantum yield is displayed in Table S 1.

*Table S 1: Relative fluorescence quantum yields of bTMS-BP, bTES-BP and bTIPS-BP with p-terphenyl as standard in cyclohexane.*

| | **bTMS-BP** | **bTES-BP** | **bTIPS-BP** |
|---|---|---|---|
| $\Phi_{Fl}$ (%) | 91 | 92 | 93 |

All measurements were corrected for their (slight) differences in absorption and concentrations were kept low to ensure that reabsorption effects do not influence the measurements (see Figure S 1).

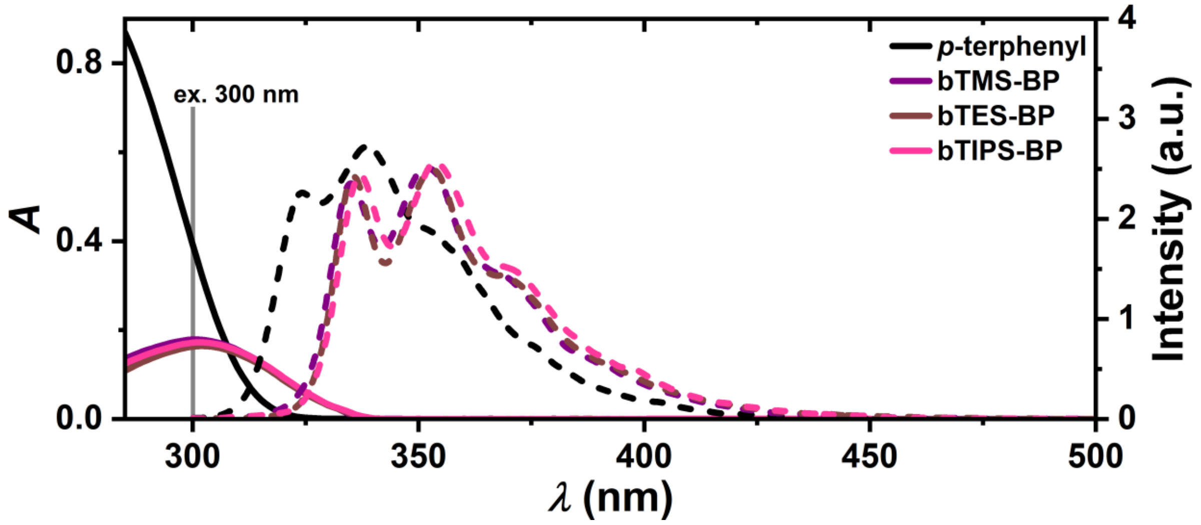


*Figure S 1: Absorption and emission spectra of 30 µM p-terphenyl, 3.5 µM_bTMS-BP, 3.5 µM bTES-BP and 3.5 µM bTIPS-BP in cyclohexane. For all emission measurements, identical settings were kept.*

These results substantiate our conclusions from the absolute quantum yield measurements that the biphenyl derivatives are highly fluorescent UVA emitters.

## 1.7 Low-intensity time-resolved upconversion emission measurements

Time-resolved photoluminescence lifetime measurements were carried out by using a time-correlated single-photon counting lifetime spectroscopy system, HAMAMATSU Quantaurus-Tau C11367-21, C11567-02 and M12977-01.

The triplet lifetime $\tau_{An}$ was extracted by the below described relationship:[8]

$$I_{UC}(t) \propto [T_{1,E}]^2 = \left\{[T_{1,E}]_0 \frac{1-\beta}{\exp(t/\tau An)-\beta}\right\}^2$$

where $I_{UC}(t)$ is the emission intensity, $[T_{1,E}]$ is the triplet concentration, $t$ is the time, and $\beta$ is the dimensionless parameter indicating the TTA efficiency ($0<\beta<1$).[9,10]

Depending on the upconversion sample, ND filters were utilized to reduce the excitation intensity, so that almost no upconversion events are present ($\beta \approx 0$). There, triplet decay through the TTA-process becomes negligible and the overall triplet decay becomes monoexponential. This is evidenced by the almost monoexponential decay of the emission of the upconversion samples displayed in Figure 4 C, where a monoexponential tail fit was used to extract the triplet lifetime.[11–14]

$$\log_{10}(I_{UC}(t)) \propto 2\log_{10}([T_{1,E}]) \propto \frac{-2}{\tau_{An}}$$

## 1.8 Time-correlated single photon counting (TCSPC) measurements

TCSPC (time-correlated single photon counting) measurements were carried out at 20°C using a mini-τ setup from Edinburgh Instruments, equipped with an Edinburgh Instruments TCC2 electronics module and an Edinburgh Instruments EPL 375 picosecond pulsed diode laser, an Edinburgh Instruments EPL 450 picosecond pulsed diode laser or an Edinburgh Instruments EPLED – 300 picosecond pulsed light emitting diode as excitation source. The resulting decay curves were globally fitted using the exponential reconvolution fit provided by the Edinburgh instruments software "Fluoracle".

## 1.9 Phosphorescence measurements

For low temperature phosphorescence measurements, a liquid nitrogen dewar was integrated into the LP980KS setup from Edinburgh Instruments. The samples in EPR tubes were placed in the dewar and cooled to 77 K using liquid nitrogen.

## 1.10 Irradiation experiments

Irradiation experiments were conducted with a 447 nm cw laser from Roithner Lasertechnik GmbH as an irradiation source. The laser power is variable between 10 mW and 1100 mW. The laser beam has a diameter of 2 x 5 mm$^2$. Based on the short continuous irradiation times (< 16 min) and low absorptivity (max. 40 % of photons, 100 µM 4CzIPN has an absorption of ~0.4 at 447 nm), no additional cooling of the samples was needed.

## 1.11 Laser flash photolysis (LFP)

All samples were prepared in 10 mm quartz glass cuvettes with a septum and a screw cap and purged with argon gas for 7 minutes before being employed. The concentration of the samples was chosen to have an optical density (OD) of < 0.2 (at the excitation wavelength) to avoid filter effects. Exact concentrations are given in the captions of the corresponding figures.

The LP980KS setup from Edinburgh Instruments equipped with an Nd:YAG Quantel Q-smart 450 laser was employed for transient absorption and time-resolved emission spectroscopy. The frequency-tripled (355 nm) output served as the excitation source. The laser pulse duration was ~5 ns and the pulse frequency was 10 Hz. The typical pulse energy used for transient absorption and emission studies was ~20 mJ unless mentioned specifically. Detection of transient absorption spectra occurred on an iCCD camera from Andor. Kinetic traces at selected wavelengths were recorded using a photomultiplier tube. The spectroscopic experiments were performed at 293 K using a cuvette holder that allows temperature control. The TA spectra were integrated over 50 ns unless otherwise specified.

## 1.12 Theoretical calculations

DFT calculations were performed with the program Orca 5.0.4 or 6.0.1.[15] The functional B3LYP and the basis set def2-SVP were used. After geometry optimizations, vibrational frequencies were calculated. No imaginary vibrational frequencies were obtained, indicating convergence on minimum structures. Geometry optimizations of the lowest triplet states ($T_1$ states) were performed starting from the energy-minimized geometries of the respective singlet ground states. Additional single-point calculations were carried out to obtain the spin densities of the optimized triplet state based on Mulliken population analysis. The structures and spin densities (iso value: 0.005) were displayed using the software Avogadro1.2.0.[16] In the depiction of spin densities, blue-colored surfaces denote positive spin densities, whereas red-colored surfaces denote negative spin densities. Adiabatic $T_1$ state energies were determined by comparing the energies of the optimized structures of the singlet ground state and $T_1$ state for each compound. To determine electronic excitation energies and oscillator strengths of the lowest 150 vertical transitions starting from the $T_1$ state, TD-DFT calculations were performed with previously geometry-optimized structures at the same level of theory.

*Table S 2: Energies of the $T_1$ and $T_2$ states along with the $T_2$-$T_1$ energy difference ($\Delta E_{T_2-T_1}$) of the annihilators under investigation (calculated by DFT, B3LYP/def2-SVP level of theory).*

| Compound | $E_{T_1}$ / eV | $E_{T_2}$ / eV | $\Delta E_{T_2-T_1}$ / cm$^{-1}$ |
|---|---|---|---|
| **bTMS-BP** | 2.40 | 3.70 | 10426 |
| **bTES-BP** | 2.46 | 3.74 | 10373 |
| **bTIPS-BP** | 2.40 | 3.67 | 10302 |
| **bTPhS-BP** | 2.40 | 3.68 | 10286 |

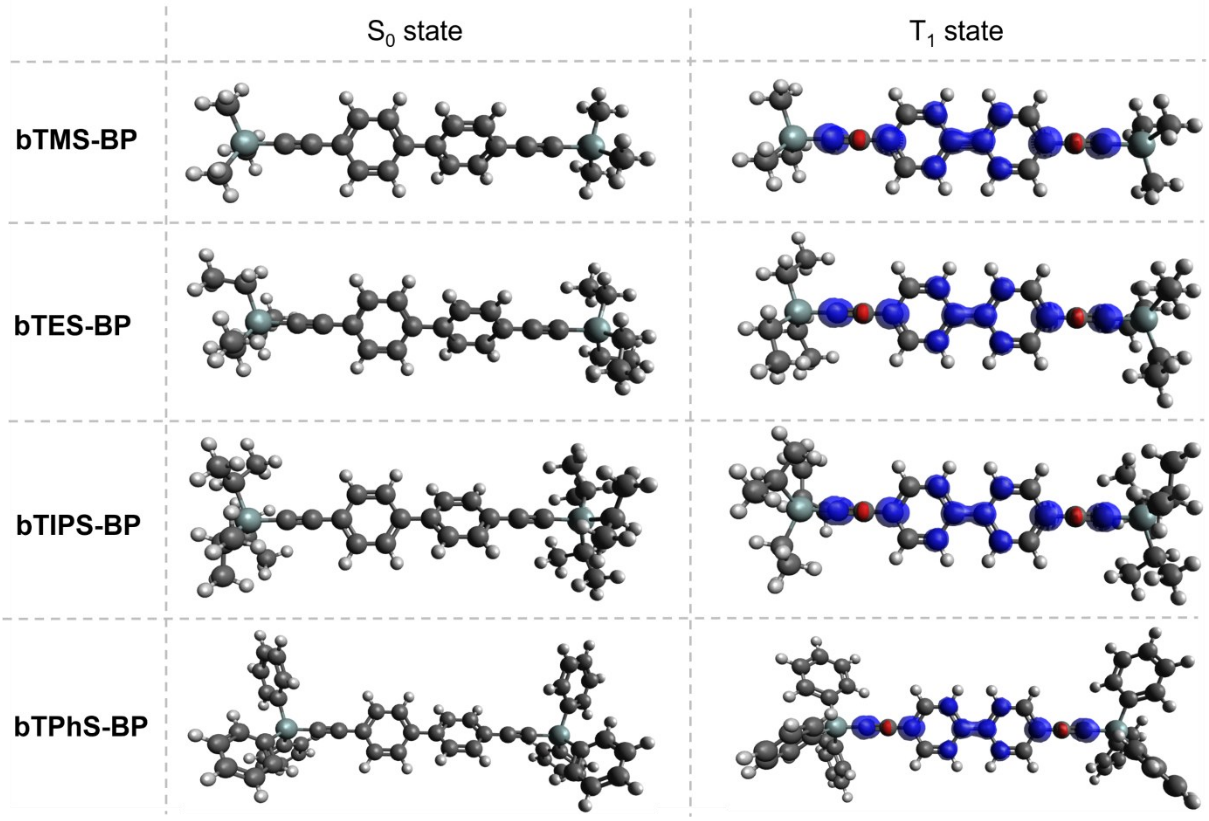


*Figure S 2: Geometry-optimized structures of the $S_0$ and $T_1$ state as well as the depiction of the spin densities of the $T_1$ state (iso value: 0.005) of the annihilators under investigation (B3LYP/def2-SVP level of theory).*

The calculated triplet energies of both, $T_1$ and $T_2$ are closely together, which manifests the experimental observation that all four compounds share almost identical photophysical properties (compare Table 1 and Figure 2 of the main part).

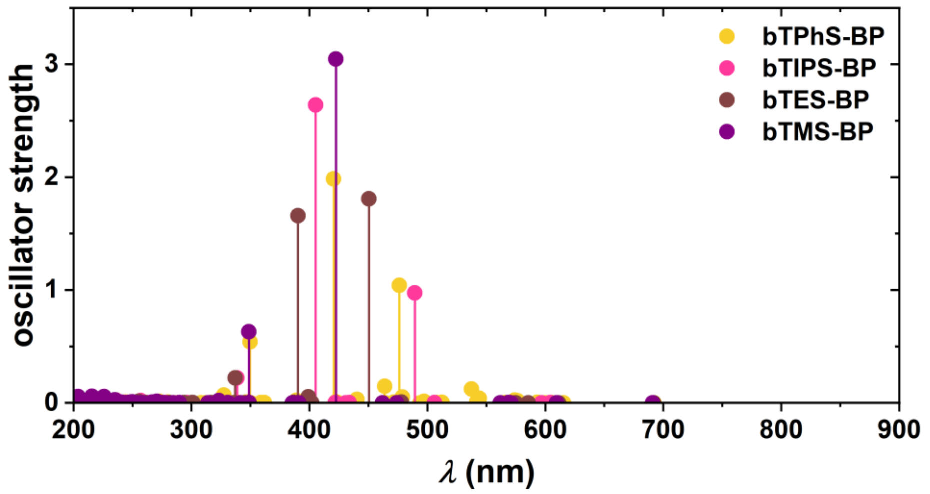


*Figure S 3: Calculated oscillator strengths of $T_1 \rightarrow T_n$ transitions of bTMS-BP, bTES-BP, bTIPS-BP and bTPhS-BP plotted against the wavelength.*

The calculated $T_1 \rightarrow T_n$ transitions show visible differences, but their general shape is similar to the experimentally found shape of the $T_1 \rightarrow T_n$ absorption spectrum. The experimental values are generally red-shifted, which might be due to the fact that the calculations are carried out for molecules in gas phase without specific solvent effects.

### 1.13 Field-desorption mass spectrometry

Low-resolution FD mass spectra were acquired using a Thermo Fisher DFS mass spectrometer equipped with an LIFDI upgrade from Linden ChroMasSpec GmbH, configured for FD mode. Ion source parameters were set as follows: acceleration voltage, +5 kV; counter electrode voltage, -5 kV; reference inlet temperature, 80°C; ion source temperature, 50°C; scan duration: 8 s; interscan delay: 200 ms. The CarboTec 10 µm allround emitter was used, operating at 75 mA. Prior to sample runs, new emitters underwent a preconditioning process involving 2 hours of heating with emitter heating current. Toluene was used as a solvent to deposit the sample onto the emitter. DFS MS scanned from m/z 100 to 1000 at a rate of 8 s/decay in low resolution mode with a m/z accuracy of ±0.5.

## 2. Synthetic procedure

Sonogashira coupling reactions were carried out for the synthesis of the biphenyl derivatives. The exact reagent loadings and yields are given for the various derivatives. A flame-dried curved two-necked flask with Liebig condenser was charged with tetrahydrofuran, 4,4'-dibromobiphenyl, bis(triphenylphosphine)palladium(II)dichloride, copper(I)iodide and triphenylphosphine under argon flow. The respective amine was added under stirring. The reaction mixture was degassed and kept under inert conditions. After heating the reaction mixture to 70°C, the corresponding silylacetylene was added dropwise over half an hour. Then the reaction mixture was stirred at reflux overnight. Afterwards, the solvent was evaporated under reduced pressure at 40°C. The product was

then redissolved in chloroform and extracted twice with water and once with brine. The organic layer was dried with $Na_2SO_4$, filtered and chloroform was removed at 40°C under reduced pressure. The product was purified by liquid column chromatography.

## 2.1 Synthesis of bTMS-BP

Educts: 1 g 4,4'-dibromobiphenyl (3.2 mmol), 51.1 mg $Pd(PPh_3)_2Cl_2$ (0.07 mmol), 52.6 mg $PPh_3$ (0.2 mmol), 41.0 mg CuI (0.2 mmol), 1.75 ml (trimethylsilyl)acetylene (14.2 mmol), 13 ml triethylamine, 28 ml dry THF.

Yield: 0.78 g, 2.25 mmol (70 %)

$^1H$ NMR (400 MHz, $CDCl_3$): δ (ppm) = 7.52 (pseudo-s, 8H); 0.26 (pseudo-s, 18H).

$^{13}C$ NMR (101 MHz, $CDCl_3$): δ (ppm) = 140.33; 132.59; 126.86; 122.56; 104.98; 95.37; 0.14.

FD-MS (m/z): calculated for $[C_{22}H_{26}Si_2]^+$: 346.16, found 346.21.

$R_f$ (cyclohexane) = 0.25

## 2.2 Synthesis of bTES-BP

Educts: 1.01 g 4,4'-dibromobiphenyl (3.2 mmol), 51.8 mg $Pd(PPh_3)_2Cl_2$ (0.07 mmol), 53.9 mg $PPh_3$ (0.2 mmol), 38.4 mg CuI (0.2 mmol), 2.2 ml (triethylsilyl)acetylene (14.2 mmol), 13 ml triethylamine, 28 ml dry THF.

Yield: 1.21 g, 2.8 mmol (88 %)

$^1H$ NMR (400 MHz, $CDCl_3$): δ (ppm) = 7.56 – 7.50 (m, 8H); 1.07 (t, 18H); 0.70 (q, 12H).

$^{13}C$ NMR (101 MHz, $CDCl_3$): δ (ppm) = 140.33; 132.67; 126.86; 122.76; 106.23; 92.87; 7.66; 4.58.

FD-MS (m/z): calculated for $[C_{28}H_{38}Si_2]^+$: 430.78, found 430.28.

$R_f$ (cyclohexane) = 0.30

## 2.3 Synthesis of bTIPS-BP

Educts: 1.01 g 4,4'-dibromobiphenyl (3.2 mmol), 51.4 mg $Pd(PPh_3)_2Cl_2$ (0.07 mmol), 55.3 mg $PPh_3$ (0.2 mmol), 42.1 mg CuI (0.2 mmol), 1.9 ml (triisopropylsilyl)acetylene (12.3 mmol), 13 ml triisopropylamine, 28 ml dry THF.

Yield: 1.07 g, 2 mmol (65 %)

$^1H$ NMR (400 MHz, $CDCl_3$): δ (ppm) = 7.58 – 7.48 (m, 8H); 1.14 (pseudo-s, 42H).

$^{13}C$ NMR (101 MHz, $CDCl_3$): δ (ppm) = 140.31; 132.66; 126.88; 122.98; 106.97; 91.81; 18.84; 11.50.

FD-MS (m/z): calculated for $[C_{34}H_{50}Si_2]^+$: 514.35, found 514.42.

$R_f$ (cyclohexane) = 0.40

## 2.4 Synthesis of bTPhS-BP

Educts: 0.5 g 4,4'-dibromobiphenyl (1.6 mmol), 25.3 mg $Pd(PPh_3)_2Cl_2$ (0.035 mmol), 16.8 mg $PPh_3$ (0.064 mmol), 12.2 mg CuI (0.064 mmol), 0.87 g (triphenylsilyl)acetylene (3.5 mmol), 7.5 ml triisopropylamine, 14 ml dry THF.

The substance was additionally purified by vapor diffusion from pentane into toluene.

Yield: 0.32 g, 0.48 mmol (30 %)

$^1$H NMR (400 MHz, $CDCl_3$): δ (ppm) = 7.74 – 7.72 (m, 12H); 7.68 – 7.57 (m, 8H); 7.46 – 7.16 (m, 18H).

$^{13}$C NMR (101 MHz, $CDCl_3$): δ (ppm) = 140.85; 135.77; 133.65; 132.94; 130.12; 128.16; 127.03; 122.27; 109.38; 90.39.

FD-MS (m/z): calculated for $[C_{52}H_{38}Si_2]^+$: 718.25, found 718.52.

$R_f$ (toluene) = 0.90

# 3. Additional spectroscopic data

## 3.1 Stern–Volmer measurements

The unquenched triplet lifetime of $Ir(dFppy)_3$ in toluene was measured to be 1.41 µs.

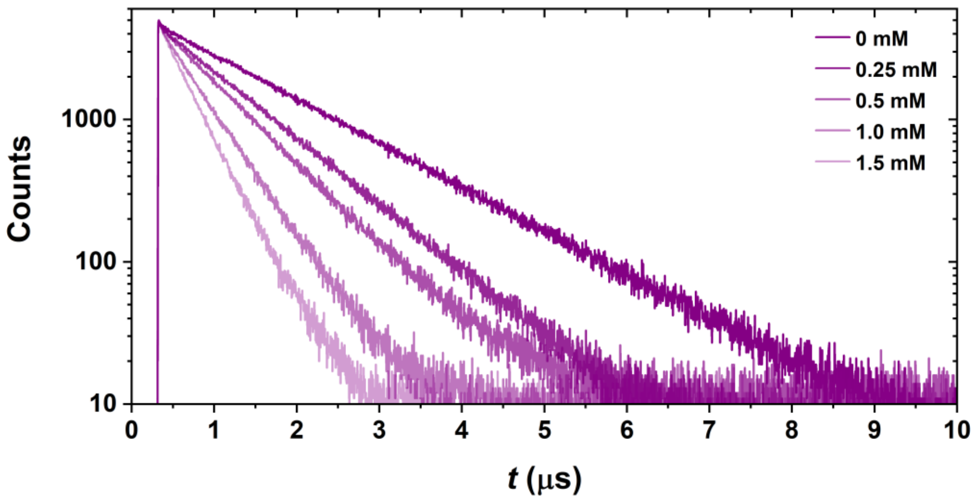


*Figure S 4: Stern–Volmer raw data for the quenching of 15 µM $Ir(dFppy)_3$ with different concentrations of bTMS-BP in degassed toluene. The kinetic decay traces were recorded with the above-described TCSPC setup. Excitation occurred at 450 nm.*

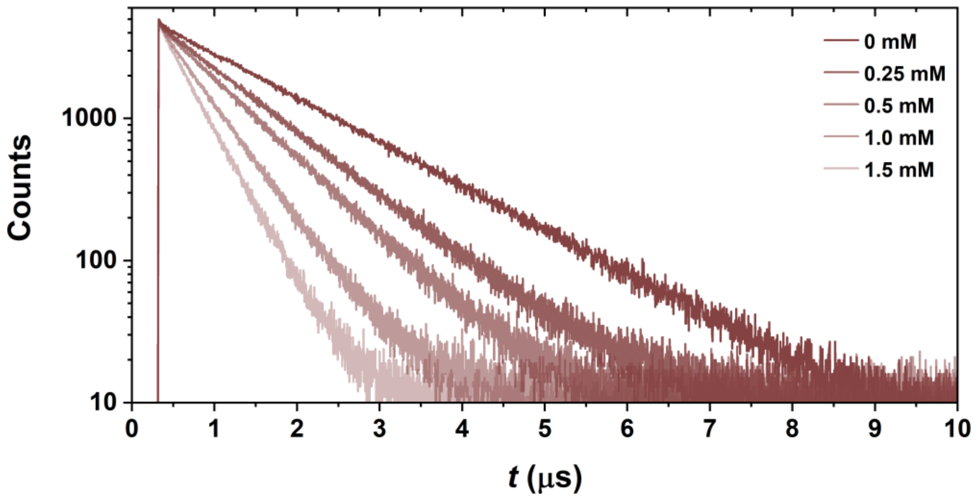


*Figure S 5: Stern–Volmer raw data for the quenching of 15 μM Ir(dFppy)₃ with different concentrations of bTES-BP in degassed toluene. The kinetic decay traces were recorded with the above-described TCSPC setup. Excitation occurred at 450 nm.*

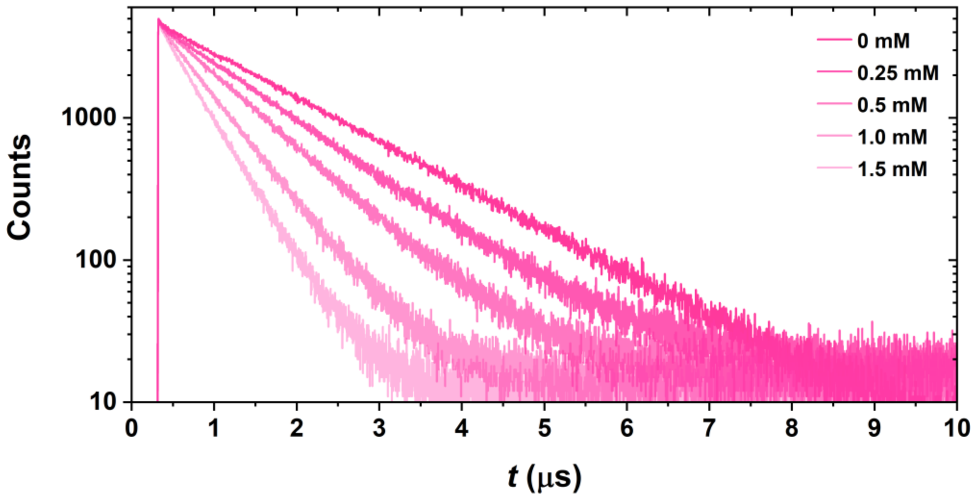


*Figure S 6: Stern–Volmer raw data for the quenching of 15 μM Ir(dFppy)$_3$ with different concentrations of bTIPS-BP in degassed toluene. The kinetic decay traces were recorded with the above-described TCSPC setup. Excitation occurred at 450 nm.*

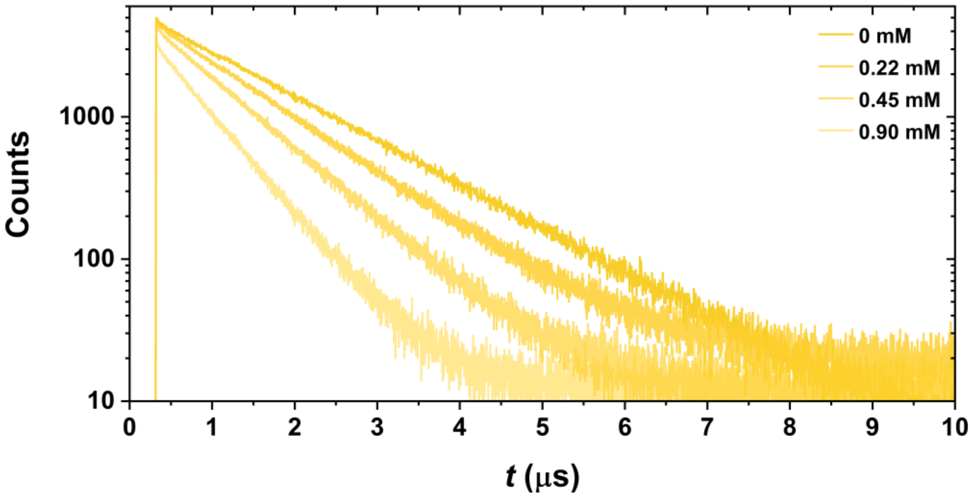


*Figure S 7: Stern–Volmer raw data for the quenching of 15 μM Ir(dFppy)₃ with different concentrations of bTPhS-BP in degassed toluene. The kinetic decay traces were recorded with the above-described TCSPC setup. Excitation occurred at 450 nm.*

## Stern Volmer experiments for 4CzIPN quenching with bTIPS-BP

The unquenched triplet lifetime of 4CzIPN in toluene was measured to be 4.75 µs.

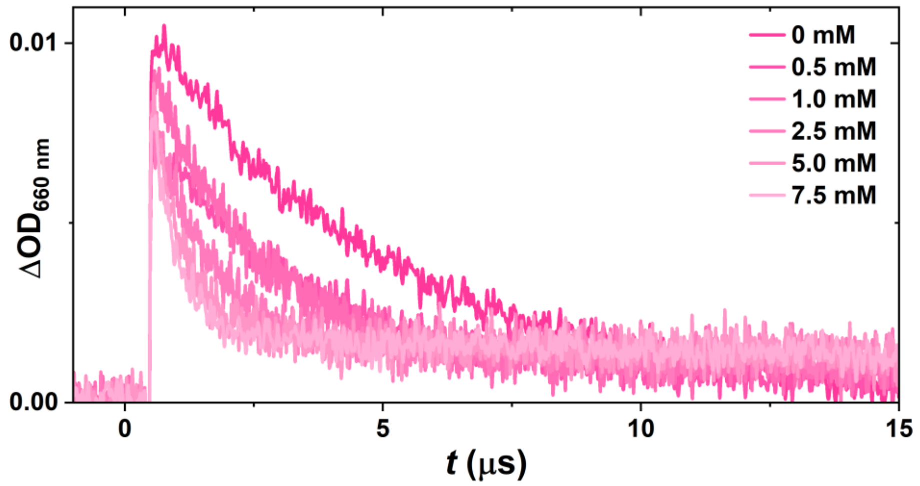


*Figure S 8: Stern–Volmer raw data for the quenching of 15 µM 4CzIPN with different concentrations of bTIPS-BP in degassed toluene. The kinetic decays were recorded with the above-described laser flash photolysis setup upon 355 nm laser excitation.*

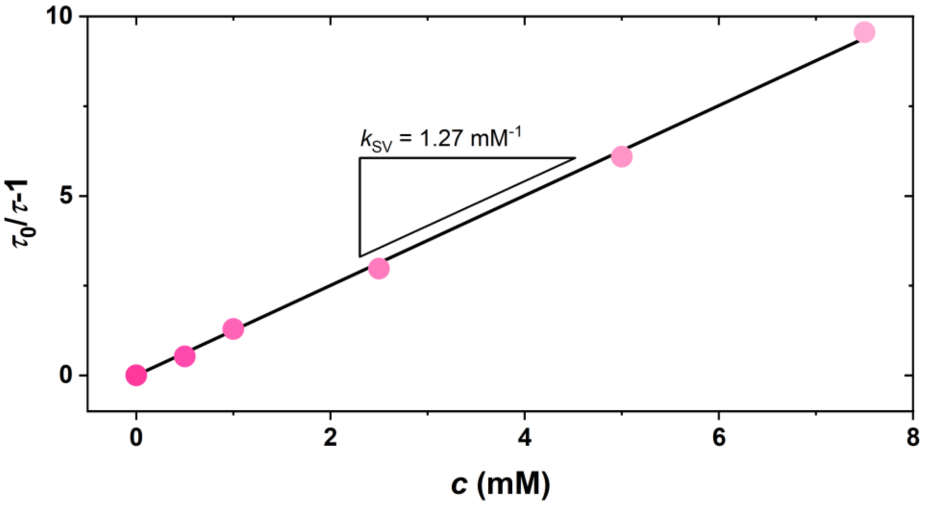


*Figure S 9: Stern–Volmer plot related to the raw date sets shown in Figure S7. The quenching rate $k_q$ amounts to $k_q = 2.68 \cdot 10^8$ M$^{-1}$ s$^{-1}$.*

## 3.2 Upconversion and CMNB-caged fluorescein activation with 4CzIPN and bTIPS-BP

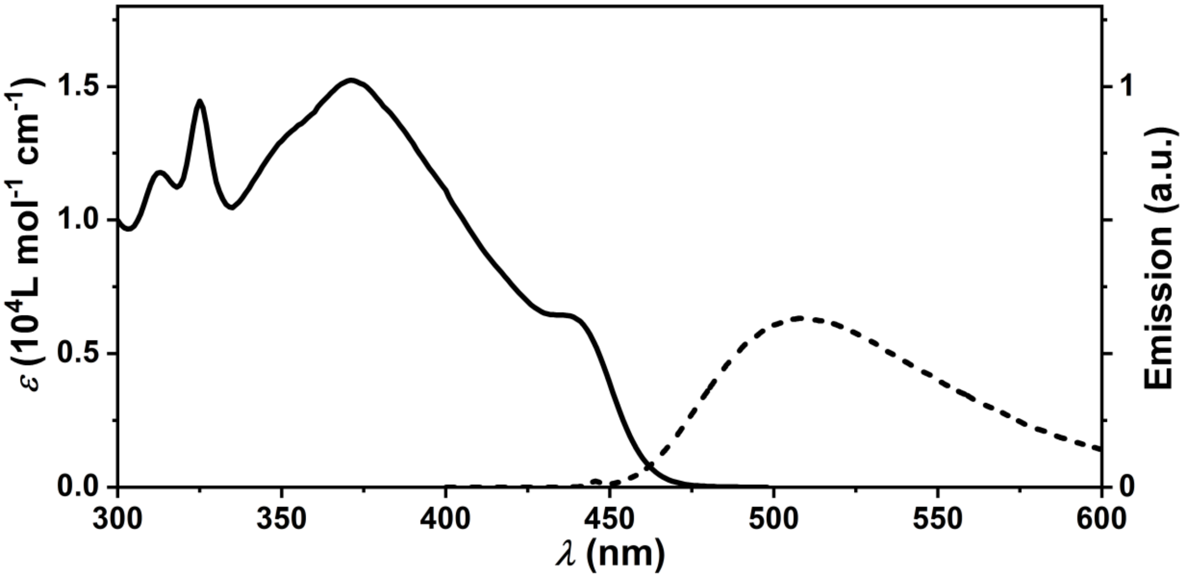


*Figure S 10: Calibrated UV-vis absorption spectrum and normalized emission spectrum of 4CzIPN in deaerated toluene.*

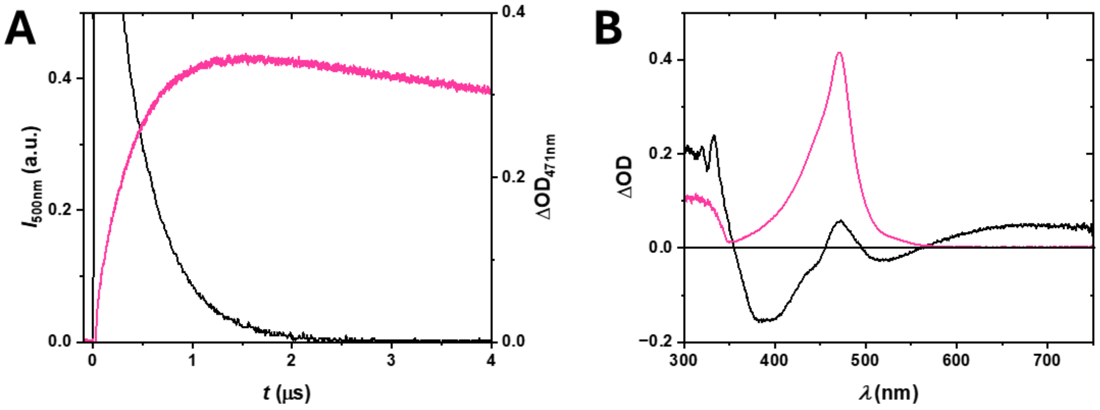


*Figure S 11: **A**: Scaled kinetic transient absorption (pink) and emission (black) decay traces of 25 µM 4CzIPN with 7 mM bTIPS-BP in toluene. **B**: Transient absorption spectra of bTIPS-BP (pink, 5 µs after excitation, 100 ns integration time) and 4CzIPN (black, 100 ns after excitation, 100 ns integration time) excited with a pulsed 355 nm laser.*

The transient absorption maximum of bTIPS-BP rises with the decay of the delayed fluorescence of 4CzIPN, which directly corresponds to the relative concentration of $^{3}$4CzIPN. Since the evolving transient absorption spectrum is identical to the $T_1$ absorption spectrum of bTIPS-BP obtained with the Ir-based sensitizer, this can be regarded as evidence for TET with the organic TADF sensitizer.

### Uncaging of fluorescein and tracking via fluorescence spectroscopy

Flourecein can either be found as a lactone or a quinoid. However, its highly fluorescent species is known to be the doubly deprotonated form (Scheme S 1).[17] In a water/DMF mixture with a ratio of 1:1, some deprotonation is ensured since DMF acts as a very weak

base.[18] The deprotonated species can than be detected utilizing fluorescence spectroscopy.

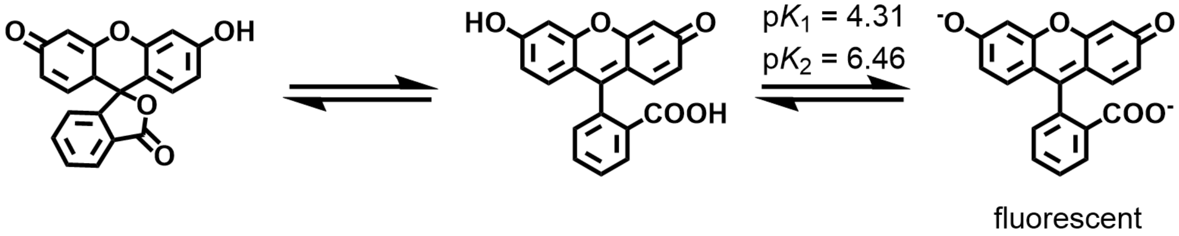


*Scheme S 1: Molecular structures and pH-dependent equilibria of fluorescein.*

As control experiment, a sample that contains 100 µM 4CzIPN in degassed toluene was irradiated with a 447 nm 1 W cw laser next to a sample containing 15 µM CMNB-caged fluorescein in $H_2O$/DMF (1:1). The setup was arranged as described in Fig. 5 **B**. After 30 minutes of irradiation, the sample containing 15 µM CMNB-caged fluorescein was irradiated with a 370 nm LED from Kessil with a 3 cm spacing.

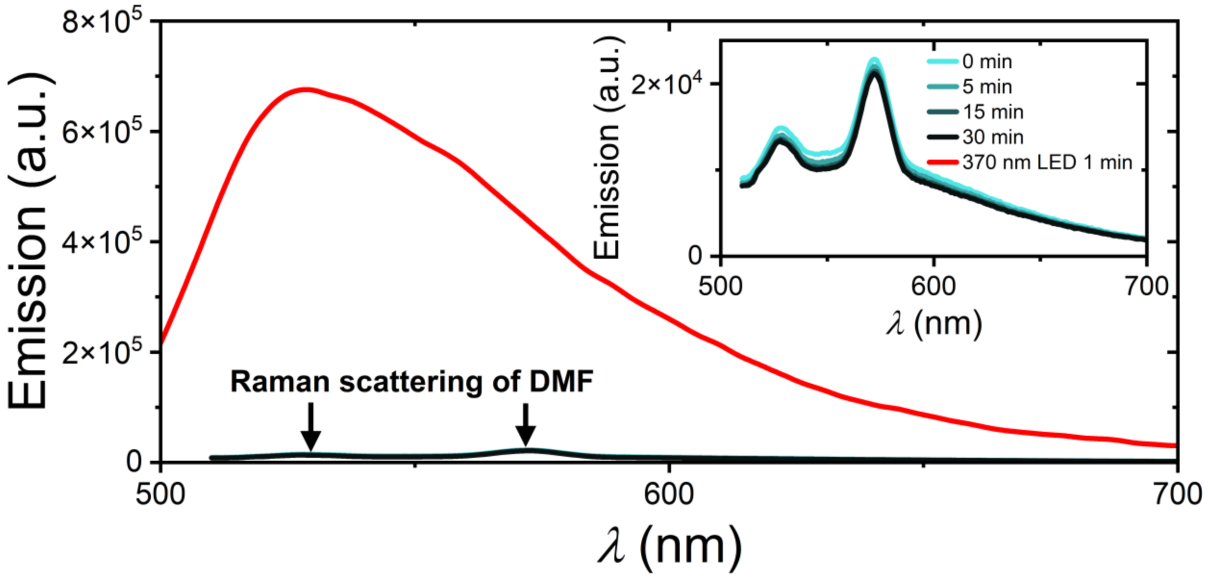


*Figure S 12: Control experiments: CMNB-caged Fluorescein excited at 490 nm after irradiation of a cuvette containing 100 µM 4CzIPN with this sample next to it. See text for further explanations. Inset: Emission signals from the main plot on enlarged scale.*

Since CMNB-caged fluorescein does not emit when excited at 490 nm, almost no emission could be detected. Intense excitation only led to the detection of Raman scattering of DMF.[19] Irradiation of a sample consisting of only 100 µM degassed 4CzIPN with a 1 W 447 nm cw laser in direct proximity to a cuvette to a sample of 15 µM CMNB-caged Fluorescein led to no conversion of CMNB-caged fluorescein to fluorescein. Irradiation with a 370 nm LED from Kessil at 3 cm distance, however, led to a direct conversion to fluorescein, which was detected by its characteristic emission (compare Figure S 12). This confirms that the uncaging reaction is driven by the upconverted emission of bTIPS-BP.

## Estimation of the efficiency of the radiative energy transfer to the substrate

To evaluate the efficiency of the energy transfer from the upconversion solution (see Figure 5 B), we roughly estimated the number of photons which are emitted from the upconversion solution in the direction of the substrate cuvette. For this, we compared the surface of the cuvette filled with photocage-containing solution to the theoretical surface of the UV irradiation area in the same distance (which can be described as the spherical surface with the distance between blue cw laser excitation and the surface of the cuvette as the radius) to give us the percentage of photons that hit the cuvette in the first place. The surface of the cuvette that can be hit with photons is estimated to be 1.85 $cm^2$ (with 3 mL of solution in the cuvette, corrected for projection on a sphere with the radius of 1.4 cm), while the total surface at this radius gives a value of 24.6 $cm^2$. Therefore, roughly 7.5 % of all emitted UV photons hit the cuvette. From these, about 70 % can be (re-)absorbed, since their emission energy overlaps with the absorption of the uncaging-substrate. The absorption of the part of the absorption spectrum, where photons from bTIPS-BP emit averages roughly out to an absorption of ~0.2 (at an optical path length of 1 cm), which means that roughly 37 % of photons in this region actually get absorbed. Combining all these rough estimates, we can assume that about 2 % of all photons generated by the upconversion system are absorbed by the photocage, underlining the high inherent efficiency of the UC system.

This estimation however, is a highly simplified approach and does not consider effects, such as varying layer thickness (and therefore filter effects) of TTA-UC pair and substrate depending on the exact photon position, as well as reflection of the emission on the cuvette and intensity dependence of the origin of photons dependent on the TTA-UC sample.

The inherent inefficiency of this setup can be overcome by the use of nanocapsules that are directly integrated in the solution of the uncaging substrate as demonstrated by Huang et al. and Congreve at al. This way, a higher percentage of photons can be absorbed, leading to an overall higher conversion efficiency.[20,21]

## 3.3 Additional photophysical properties of the annihilators

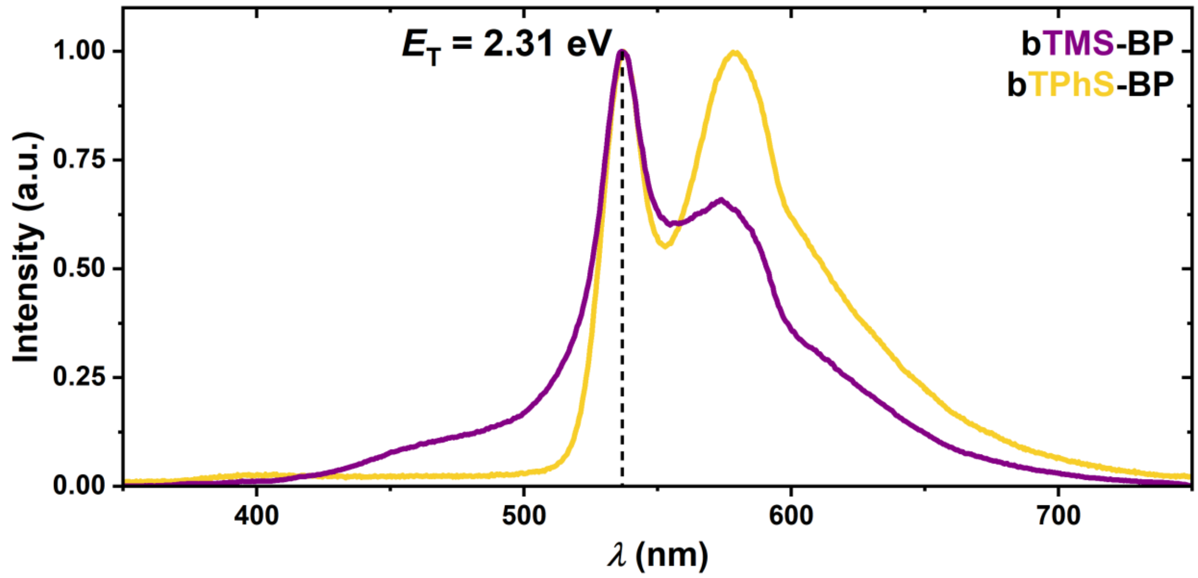


*Figure S 13: Phosphorescence spectra of bTMS-BP and bTPhS-BP at 77 K in 2-methyl-tetrahydrofuran. The spectra were recorded using the laser flash photolysis setup described above with the modifications described in the section "phosphorescence measurements". Meaningful 77 K phosphorescence spectra could not be obtained for bTES-BP and bTIPS-BP.*

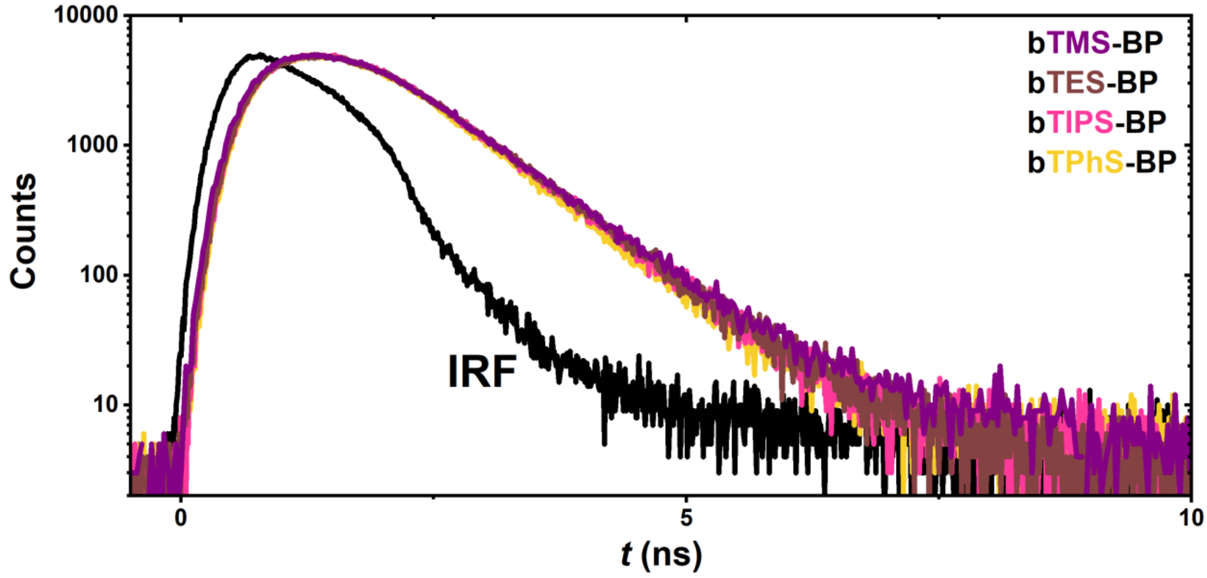


*Figure S 14: TCSPC measurements of bTMS-BP, bTES-BP, bTIPS-BP and bTPhS-BP in toluene after direct excitation using a 300 nm EPLED.*

## 3.4 Upconversion with 445 nm as excitation source

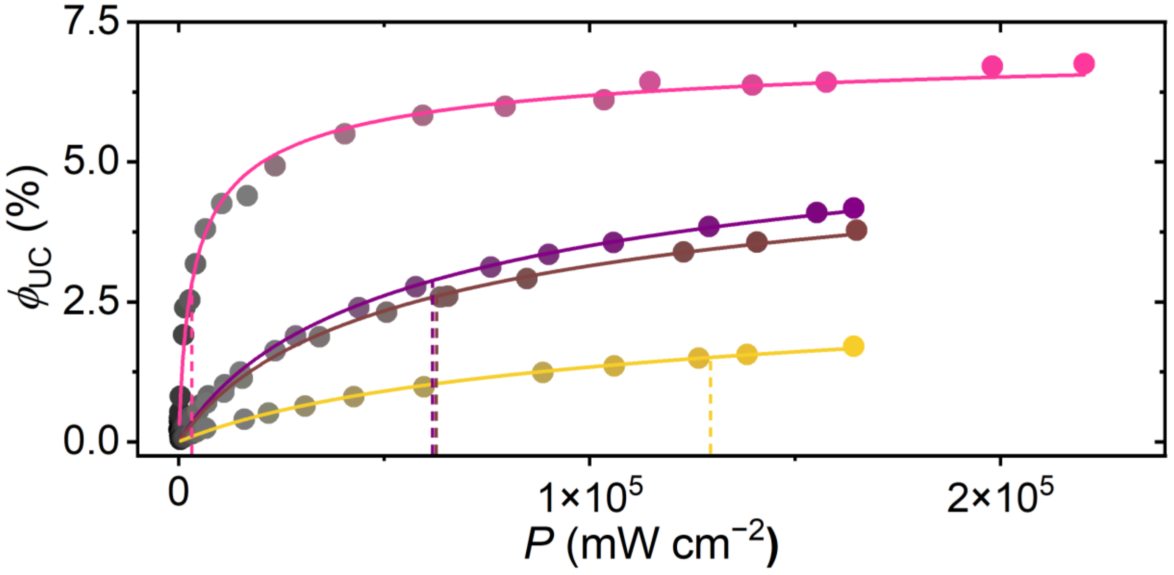


*Figure S 15: Upconversion quantum yield plotted against the excitation intensity. The upconversion sample contains 50 µM Ir(dFppy)$_3$ and 5 mM bTXS-BP in deaerated toluene. The results were fitted using the formula from Murakami and Kamada.*[6]

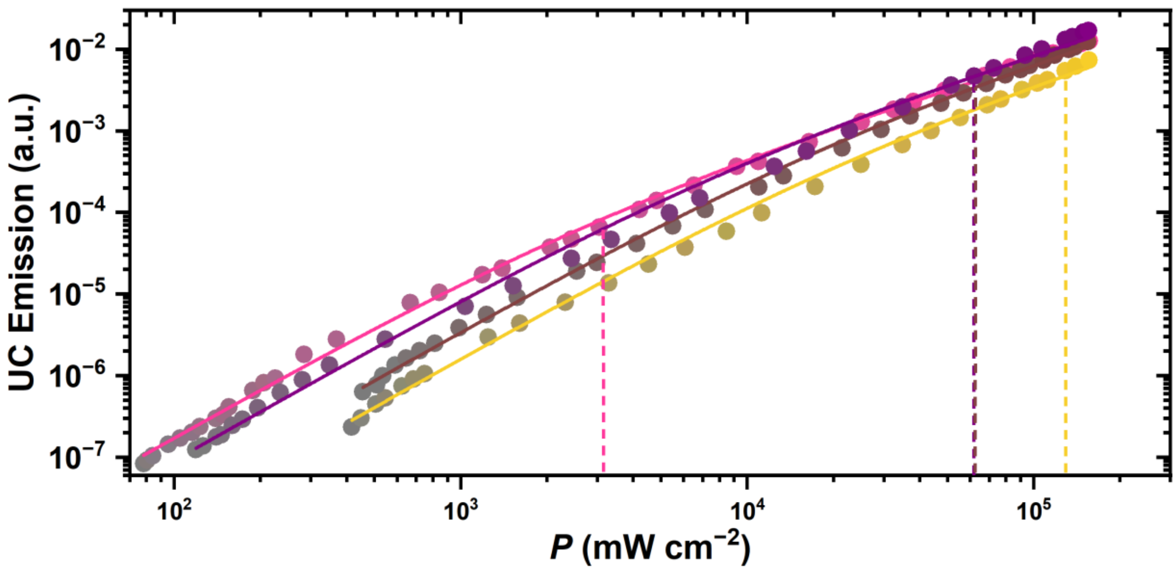


*Figure S 16: Data sets from Figure S 15 plotted in a double logarithmic fashion.*

*Table S 3: Upconversion results (445 nm excitation) of solutions comprised of 50 µM Ir(dFppy)$_3$ and 5 mM bTXS-BP in deaerated toluene presented in Figure S 15 and Figure S 16 are shown.*

| **5 mM, 445 nm** | **bTMS-BP** | **bTES-BP** | **bTIPS-BP** | **bTPhS-BP** |
|---|---|---|---|---|
| $\phi_{UC, ext.}$ (%) | 4.2 | 3.8 | 6.8 | 1.7 |
| $\phi_{UC, \infty}$ (%) | 7.5 | 6.8 | 7.4 | 3.9 |
| $I_{th}$ (W cm$^{-2}$) | 61.8 | 62.7 | 3.1 | 129.4 |

Compared to excitation with 405 nm, the upconversion quantum yields are reduced and the threshold intensities are increased. For the threshold intensity, this is expected, since the same excitation intensity leads to less absorption and less annihilator molecules in the triplet excited state. The lower measured upconversion quantum yield is an artifact of the specific upconversion measurement setup: The emission is measured on the same side of the sample, where the excitation beam hits the cuvette. For 445 nm excitation, this leads to an increased penetration depth and stronger reabsorption effects, which reduce the measured upconversion quantum yield.

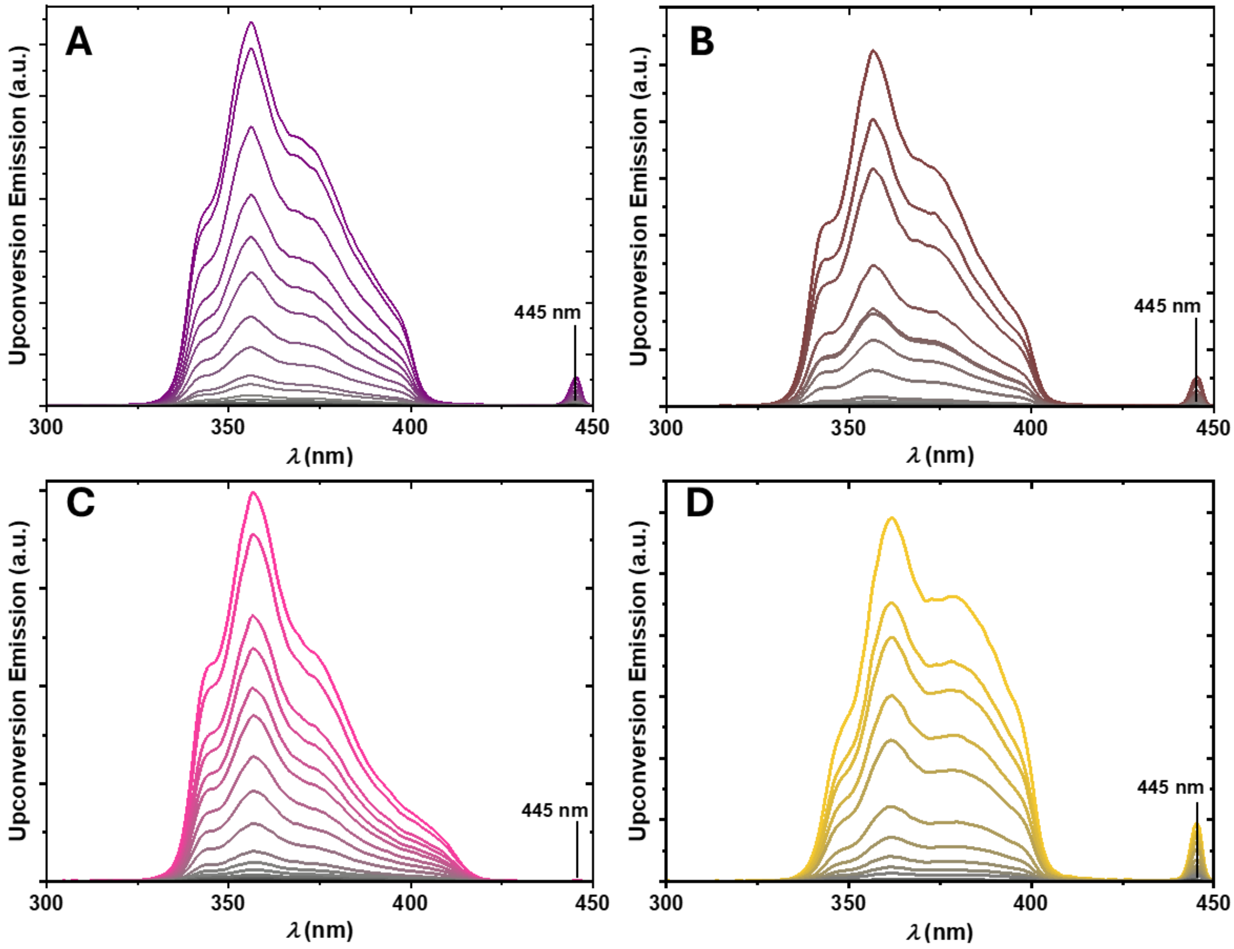


*Figure S 17: Upconversion emission spectra of 50 μM Ir(dFppy)$_3$ with **A** 5 mM bTMS-BP, **B** 5 mM bTES-BP, **C** 5 mM bTIPS-BP and **D** 5 mM bTPhS-BP in deaerated toluene. The samples were excited at 445 nm and a 425 nm shortpass filter was placed between the sample and the detector.*

## 3.5 Upconversion emission correction (405 nm excitation)

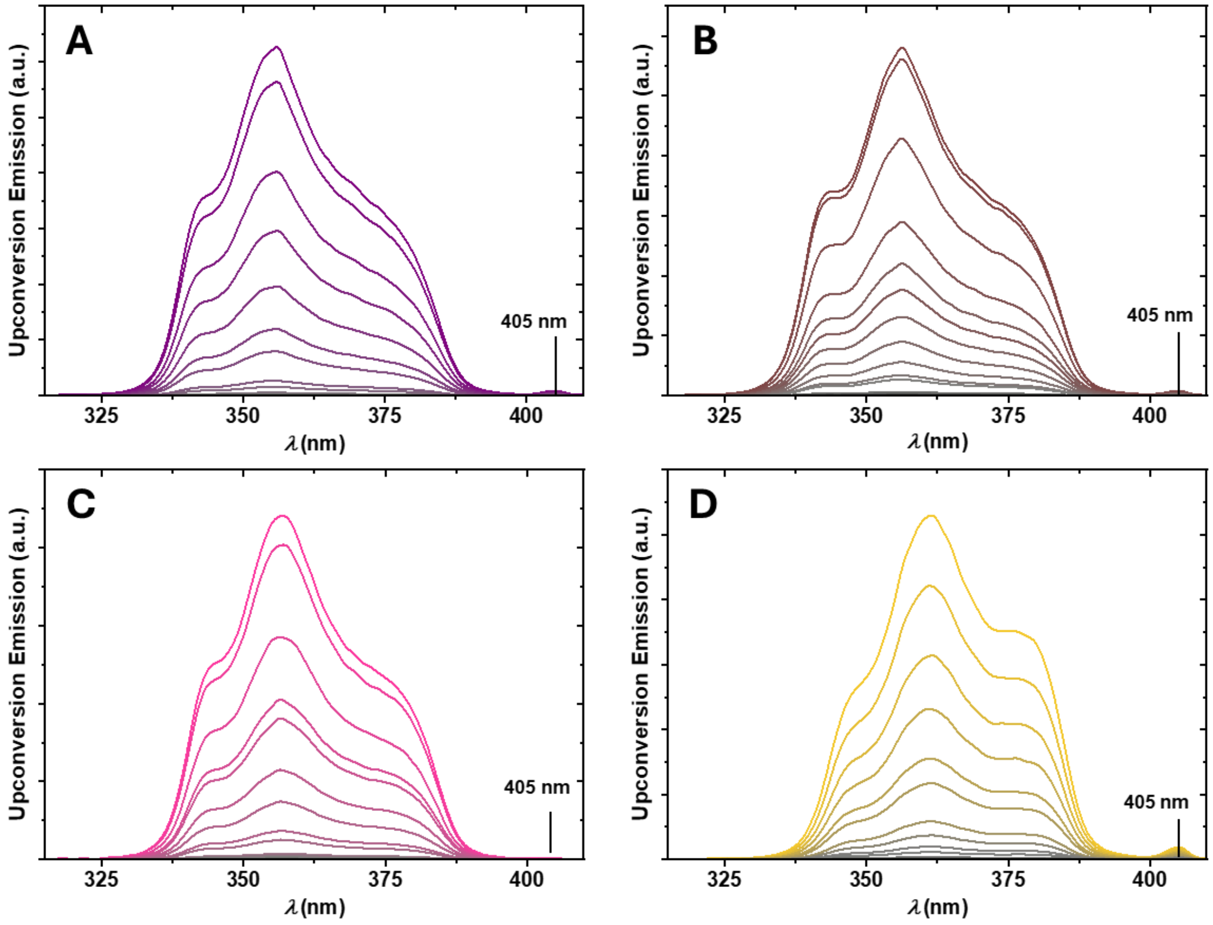


*Figure S 18: Upconversion emission spectra of 50 µM Ir(dFppy)₃ with **A** 5 mM bTMS-BP, **B** 5 mM bTES-BP, **C** 5 mM bTIPS-BP and **D** 5 mM bTPhS-BP in deaerated toluene. The samples were excited at 405 nm and a 400 nm shortpass filter was placed between the sample and the detector.*

Reabsorption effects of the annihilator were already corrected in our quantum yield analysis given that the actual emission quantum yield of the annihilators at 5 mM concentration were used. However, reabsorption effects of the sensitizer cannot be directly corrected with our experimental results. The following comparison highlights the different filter effects that can be expected with both sensitizers under study. At similar absorption in the upconverted region, 4CzIPN absorbs more photons (by a factor of 3.2), leading to a lower threshold intensity (Figure S 19). Combined with the slightly more advantageous absorption spectrum, this leads to similar upconversion quantum yields despite the reduced intersystem crossing quantum yield of 4CzIPN when compared to Ir(dFppy)$_3$.

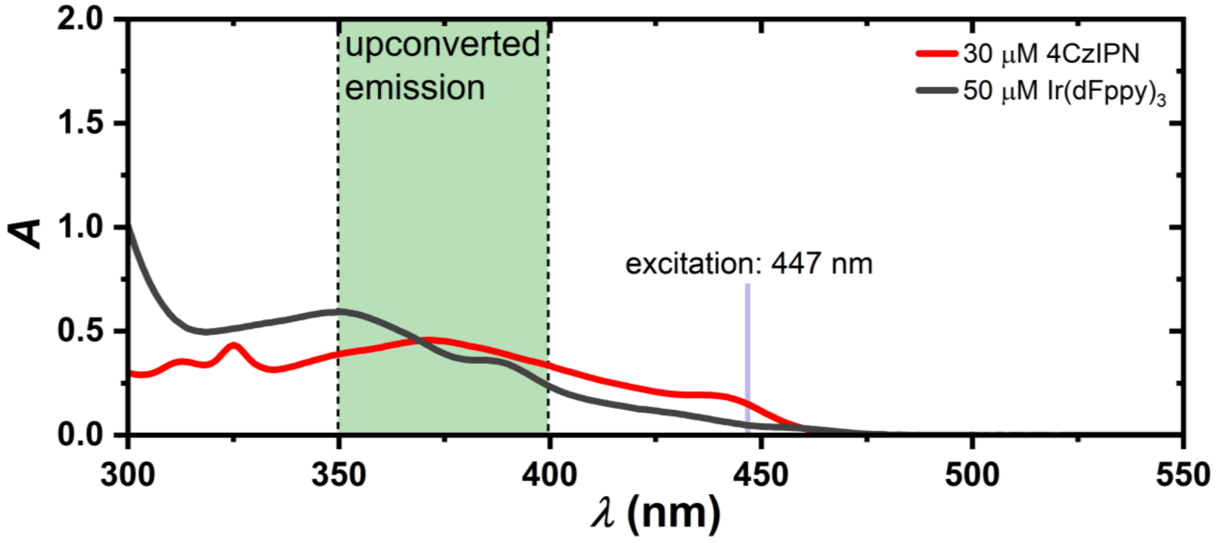


*Figure S 19: Absorption spectra of 30 µM 4CzIPN and 50 µM Ir(dFppy)$_3$ in a 1 cm cuvette in toluene. The blue line marks the excitation wavelength of the 4CzIPN upconversion system (447 nm) and the green region marks the emission region of the biphenyl derivatives.*

The experimental setup for measuring upconversion quantum yield utilizes a 400 nm shortpass filter which cuts off the low energy edge of the emission spectrum. For this, the upconverted emission can be corrected as visualized in Figure S 20. The resulting corrected upconversion quantum yields $\phi_{UC, \infty, corr.}$ And $f$-factors $f_{corr.}$ can be found in Table S 4.

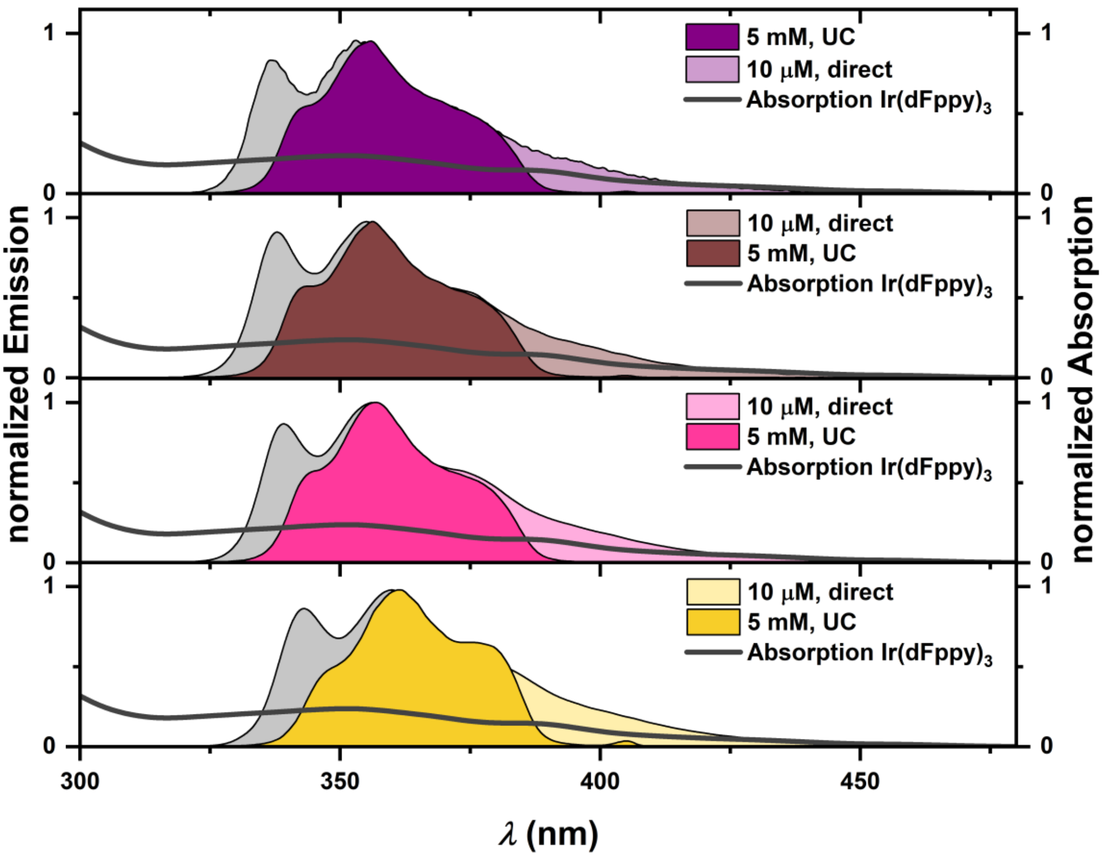


*Figure S 20: Emission spectra of bTXS-BP under direct excitation conditions and upconversion conditions. The upconversion spectra were recorded with a 400 nm shortpass filter between the sample and the detector. The gray region marks the not additionally corrected part of the emission spectrum, while the lightly colored region highlights the effect of the shortpass filter on the upconverted emission (strongly colored).*

*Table S 4: Upconversion results of solutions comprised of 50 µM Ir(dFppy)$_3$ and 5 mM bTXS-BP in deaerated toluene (405 nm excitation) and in Figure S 20 corrected for effects of the shortpass filter.*

| **5 mM, 405nm** | **bTMS-BP** | **bTES-BP** | **bTIPS-BP** | **bTPhS-BP** |
|---|---|---|---|---|
| correction factor | 1.18 | 1.20 | 1.25 | 1.24 |
| $\phi_{UC,\infty,corr.}$ (%) | 13.9 | 13.7 | 14.2 | 6.8 |
| $f_{corr.}$ | 23.8 | 25.9 | 25.5 | 14.5 |

## 3.6 Stability measurements

Upconversion systems consisting of 5 mM annihilator and 50 µM Ir(dFppy)$_3$ in toluene were prepared as described in Section 1.5 and their long-term photostability was evaluated with the same setup. A sample consisting of only 50 µM Ir(dFppy)$_3$ in toluene was measured as a reference. All samples were irradiated with the above-described 405 nm laser by RGB photonics with an excitation intensity of 93.1 W cm$^{-1}$ and a total

output power of 125 mW. Their emission spectra were collected with a cut off at 385 nm utilizing a short-pass filter for the upconversion samples. The respective spectra were integrated over their emission and normalized to yield the normalized emission found in Figure S 21.

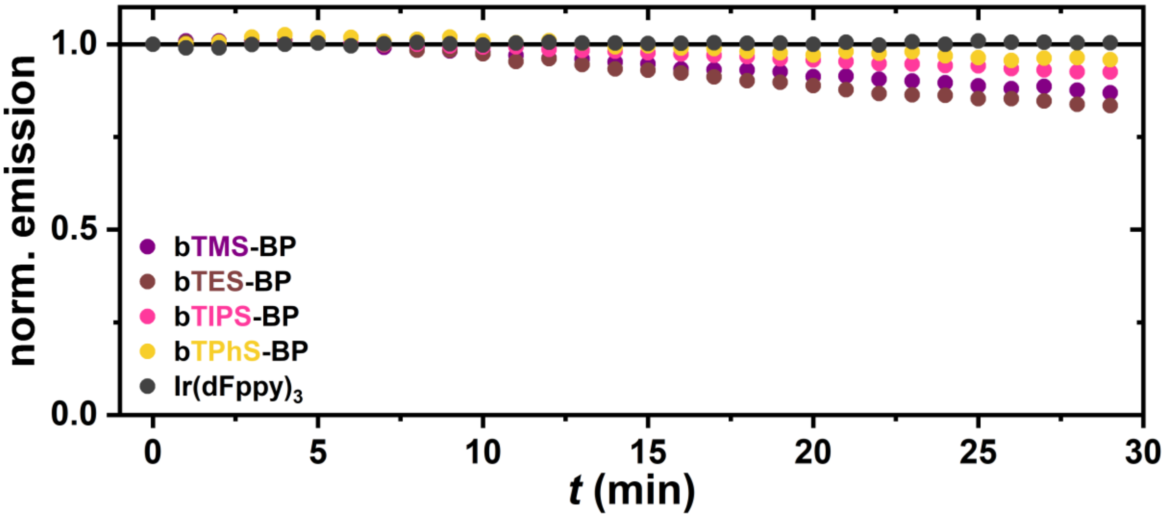


*Figure S 21: Normalized emission of samples containing 5 mM annihilator and 50 µM Ir(dFppy)₃ or only 50 µM Ir(dFppy)₃ in toluene upon 405 nm excitation plotted against the irradiation time. Exact specifications can be found in the paragraph above.*

After 30 minutes, all systems retained over 80 % of their original upconversion intensity. $Ir(dFppy)_3$ shows no visible degradation over 30 minutes of irradiation. Considering the high excitation intensity, these systems can be regarded as rather stable, when compared to other UC systems emitting in the (high-energy) UV region.

Additionally, the stability of upconversion systems under excitation of the irradiation setup (see Section 1.10) was evaluated. For this, the sample was excited with a total output power of 1100 mW of the 447 nm cw laser by Roithner and their emission at a specific wavelength was measured. The results are displayed in Figure S 22.

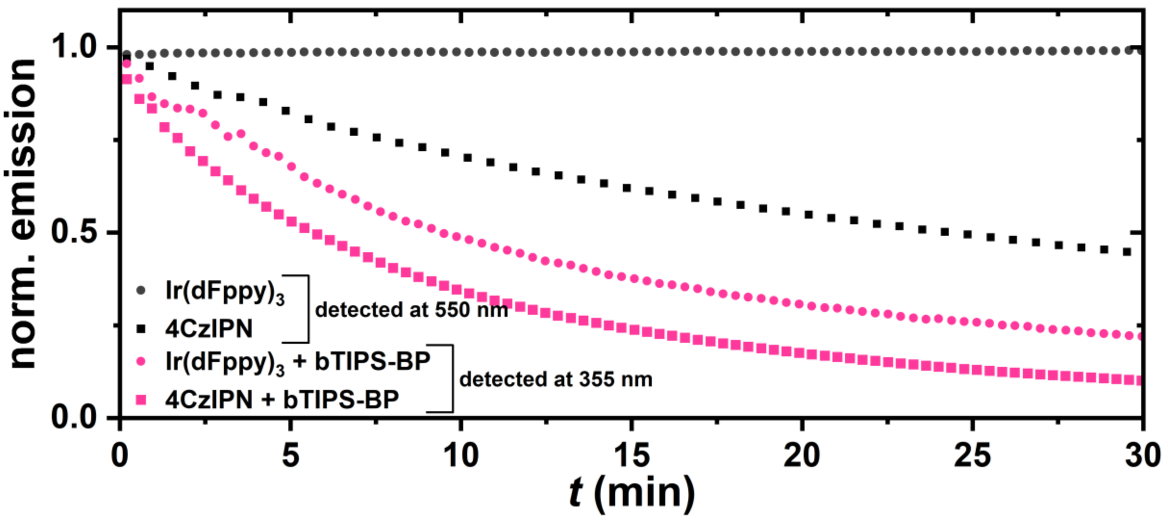


*Figure S 22: Normalized emission of samples containing 25 µM 4CzIPN with(out) 5 mM bTIPS-BP in toluene and 130 µM Ir(dFppy)$_3$ with(out) 5 mM bTIPS-BP upon 447 nm excitation plotted against the irradiation time. Exact specifications can be found in the paragraph above.*

Concentrations of 4CzIPN and Ir(dFppy)$_3$ were chosen to absorb the identical amount of photons when excited at 447 nm. While Ir(dFppy)$_3$ itself is extremely stable, the combination with bTIPS-BP leads to degradation. The rate of degradation is significantly higher compared to the results displayed in Figure S 21 because the utilized laser has a nearly 10-fold increased total output power, leading to accelerated degradation. 4CzIPN itself is already highly photolabile. In combination with bTIPS-BP this degradation is further accelerated.

## 3.7 Excimer formation

Figure S 23 (left) confirms the annihilation nature of the triplet state of bTMS-BP: At low excitation intensities, low amounts of $^3$bTMS-BP are formed which are too short lived to undergo efficient TTA at low concentrations. Therefore, the triplet lifetime of bTMS-BP is only dependent on its natural decay rate, leading to a monoexponential decay. At higher excitation intensities, higher concentrations of $^3$bTMS-BP are formed. Here, TTA is not negligible, and the decay receives a biexponential component, where the influence decreases over time (with declining triplet concentration).

Figure S 23 (right) shows non-normalized transient absorption decay traces of solutions containing different ground-state concentrations of bTMS-BP. The excitation intensity was adjusted to yield an identical triplet concentration of $^3$bTMS-BP regardless of the bTMS-BP concentration. Since $^3$bTMS-BP decays with identical rates, independent of the ground-state concentration, no excimer formation between $^3$bTMS-BP and bTMS-BP is expected.[22]

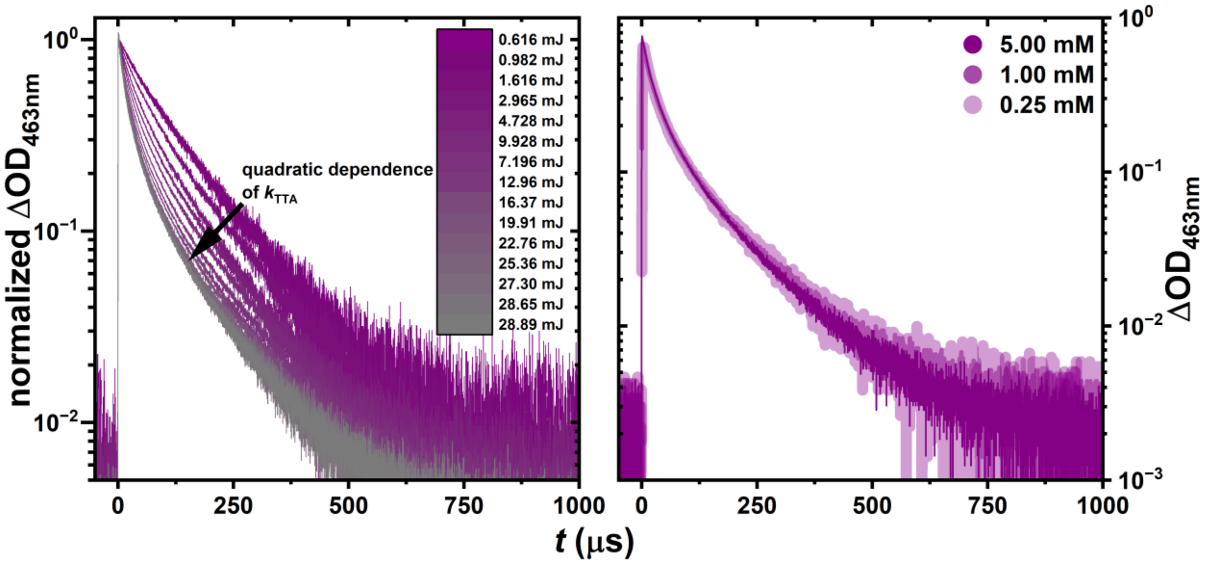


*Figure S 23: Left: Normalized kinetic absorption decay traces of a sample containing 30 µM Ir(dFppy)₃ and 5 mM bTMS-BP in toluene at different excitation intensities. Right: Kinetic absorption decays of samples containing 30 µM Ir(dFppy)₃ and 0.25, 1, 5 mM bTMS-BP in toluene. The laser excitation intensities of the measurements were adjusted to yield identical post-pulse optical densities at the absorption maximum.*

For the transient absorption spectrum, no significant changes can be detected at a delay time, where no sensitizer signal can be detected anymore. Since the energy transfer is relatively slow at lower concentrations, a delay time of 30 µs was chosen. Overall, these results confirm that no excimer formation for the sterically least demanding annihilator could be found, which is why we expect no excimer formation for all other derivatives.

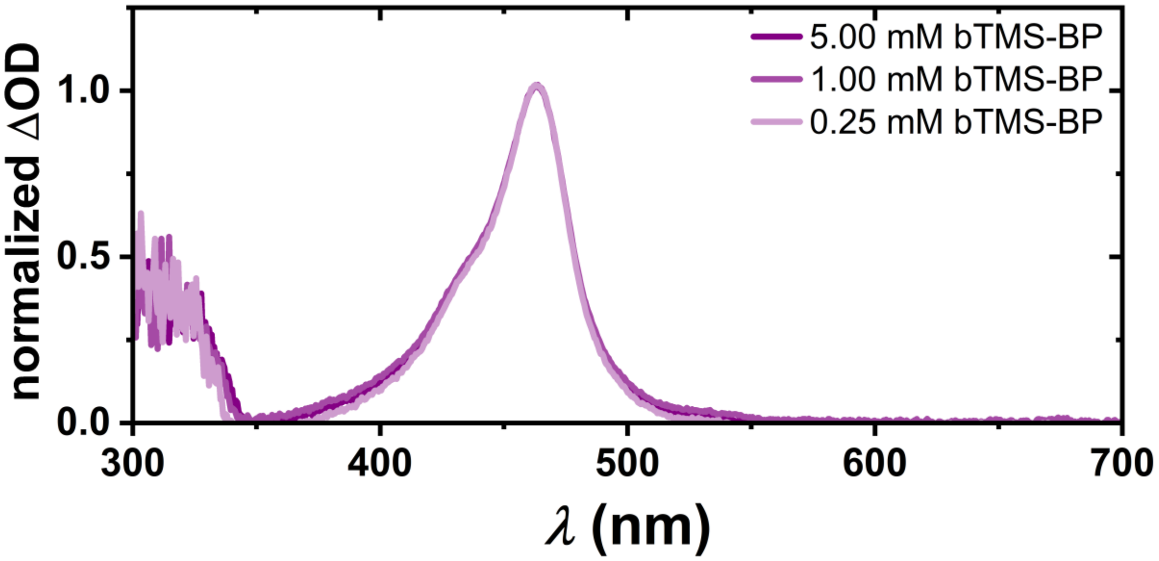


*Figure S 24: Normalized transient absorption spectra of a sample containing 30 µM Ir(dFppy)₃ and different concentrations of bTMS-BP in toluene. All spectra were taken with a 30 µs delay and integrated over 100 ns to ensure that no residual signal of Ir(dFppy)₃ is visible anymore.*

## 3.8 Triplet Annihilation Rate of bTMS-BP, bTIPS-BP and bTPhS-BP

The threshold intensity $I_{th}$ does not only depend on the unquenched triplet lifetime $\tau_0$ (or $1/ k_0^T$) of an annihilator but also on the triplet-triplet annihilation rate $k_{TTA}$ as expressed in the following equation (adapted from Meinardi et al.):[23]

$$I_{\mathrm{th}} \sim \frac{(k_0^T)^2}{k_{\mathrm{TTA}}}$$

Kinetically this is expressed in the triplet excited state concentration of annihilators in solution, as summarized by Albinsson et al. and can be measured utilizing transient absorption spectroscopy:[24]

$$\left[{}^3E^*(t)\right] = \left[{}^3E^*\right]_0 \frac{1-\beta}{\exp\left(\frac{t}{\tau_0}\right)-\beta}$$

Where $\beta$ gives the ratio between annihilator molecules in the triplet state that decay via triplet-triplet annihilation relative to the decay through all possible decay processes. Such fitted curves for raw data sets with high triplet concentration (ensured through higher laser intensities) can be found in Figure S 25.

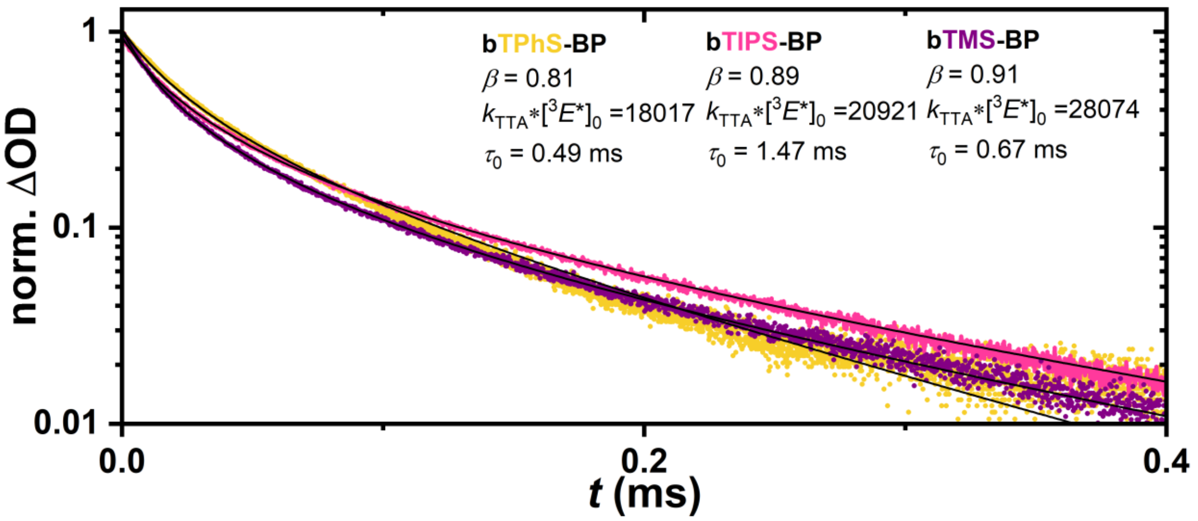


*Figure S 25: Normalized kinetic absorption decay traces of 15 µM $Ir(dFppy)_3$ with 5 mM bTMS-BP, 15 µM $Ir(dFppy)_3$ with 5 mM bTIPS-BP and 15 µM $Ir(dFppy)_3$ with 5 mM bTPhS-BP in toluene at an excitation intensity of 28 mJ per 355 nm laser pulse. Data was fitted as described by Albinsson et al.*[24]

Ignoring back-ET (which does not occur in our system), this can be expressed with the following equation:

$$\beta = \frac{2k_{\mathrm{TTA}}\left[{}^3E^*\right]_0}{k_0^T + 2k_{\mathrm{TTA}}\left[{}^3E^*\right]_0}$$

To accurately determine $k_{\mathrm{TTA}}$, one has to determine the triplet annihilator concentration at $t$ = 0. With a pulsed laser setup, this is not straightforward as additional actinometry measurements or time-consuming determinations of molar absorption coefficients for

triplets would be required, which is why we only compare the product $k_{\mathrm{TTA}} \times [{}^{3}E^{*}]_{0}$ between bTMS-BP, bTIPS-BP and bTPhS-BP. $[{}^{3}E^{*}]_{0}$ depends on the intensity of the excitation source and the energy transfer efficiency when using a standardized sensitizer concentration. If both values are identical then the difference in $k_{\mathrm{TTA}}$ can be evaluated. In this experiment, annihilator concentrations and excitation intensities were adjusted to give similar values, which should give a semi-quantitative evaluation of $k_{\mathrm{TTA}}$, where this rate constant for bTMS-BP seems to be 50% higher compared to that estimated for bTPhS-BP.

When inserting the relative $k_{\mathrm{TTA}}$ values of all three compounds, combined with the measured (relative) triplet lifetimes into the $I_{\mathrm{th}}$ relation shown above, $I_{\mathrm{th}}$ is predicted to be about 2.91 times higher for bTPhS-BP when compared to $I_{\mathrm{th}}$ for bTMS-BP, which is comparable to the 2.68 factor found in our experimental data. The predicted $I_{\mathrm{th}}$ of bTMS-BP is also calculated to be 4.84 times higher than that of bTIPS-BP, which also compares well to the experimentally found value of 4.63 These calculations thus substantiate the estimated (relative) difference in $k_{\mathrm{TTA}}$ for the three annihilators analyzed in Figure S 25 and provide the insight that for low threshold intensities, long triplet lifetimes are essential.

# 4. NMR and mass spectra

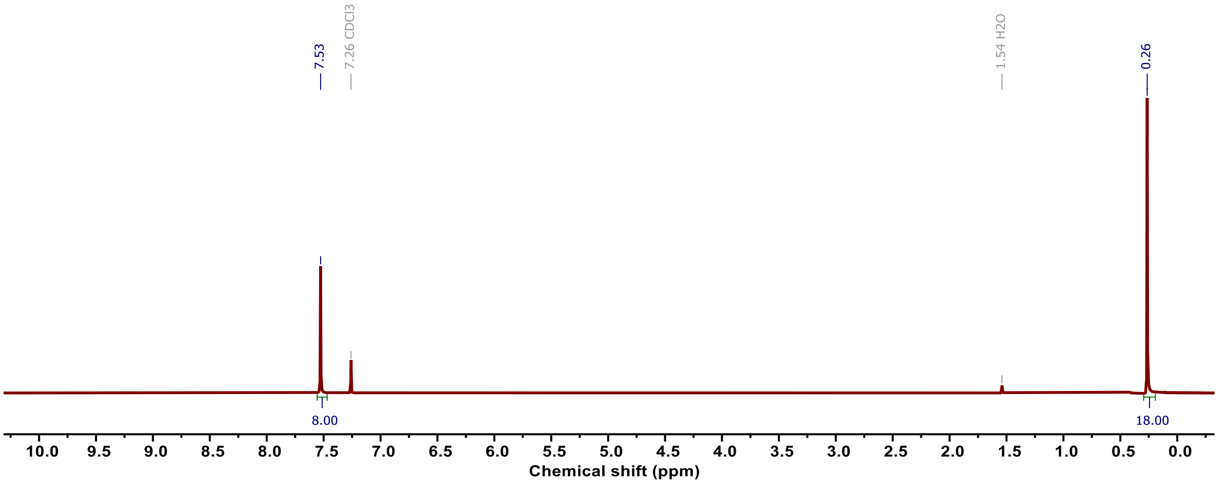


*Figure S 26: $^{1}$H NMR spectrum of bis(trimethylsilylacetylene)-biphenyl in $CDCl_3$.*

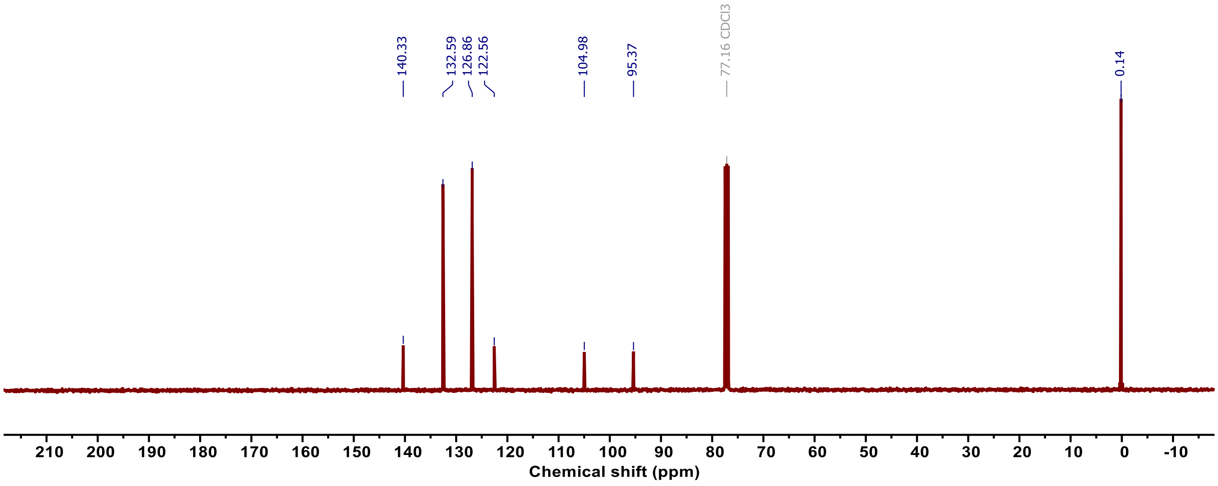


*Figure S 27: $^{13}$C NMR spectrum of bis(trimethylsilylacetylene)-biphenyl in $CDCl_3$.*

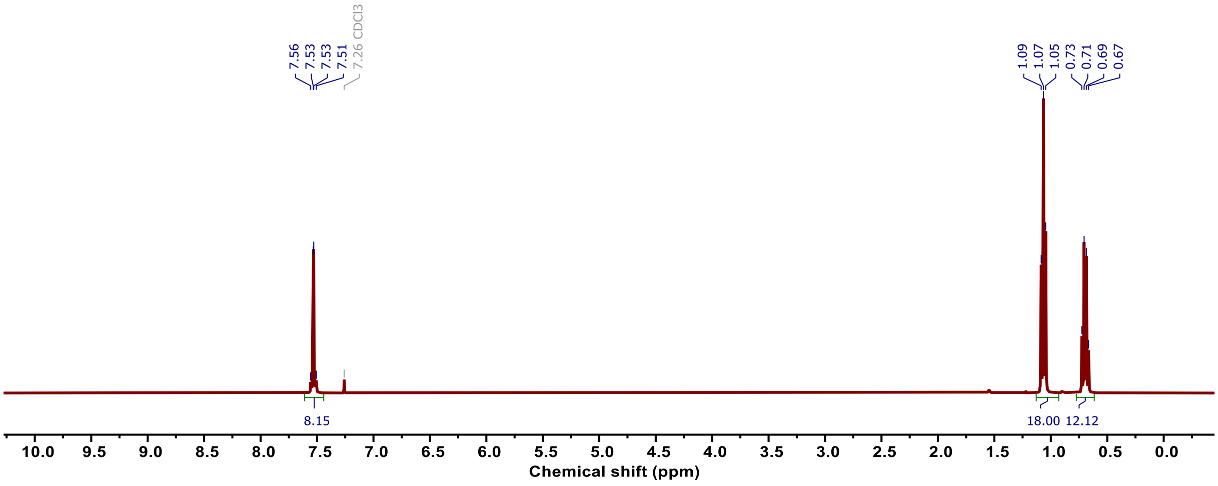


*Figure S 28: $^{1}$H NMR spectrum of bis(triethylsilylacetylene)-biphenyl in $CDCl_3$.*

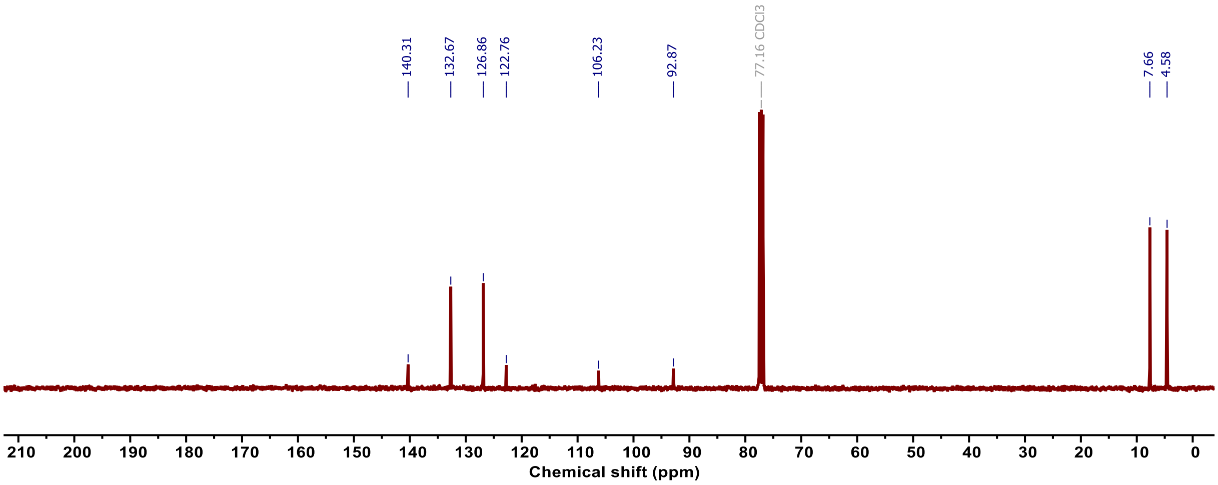


*Figure S 29: $^{13}$C NMR spectrum of bis(triethylsilylacetylene)-biphenyl in $CDCl_3$.*

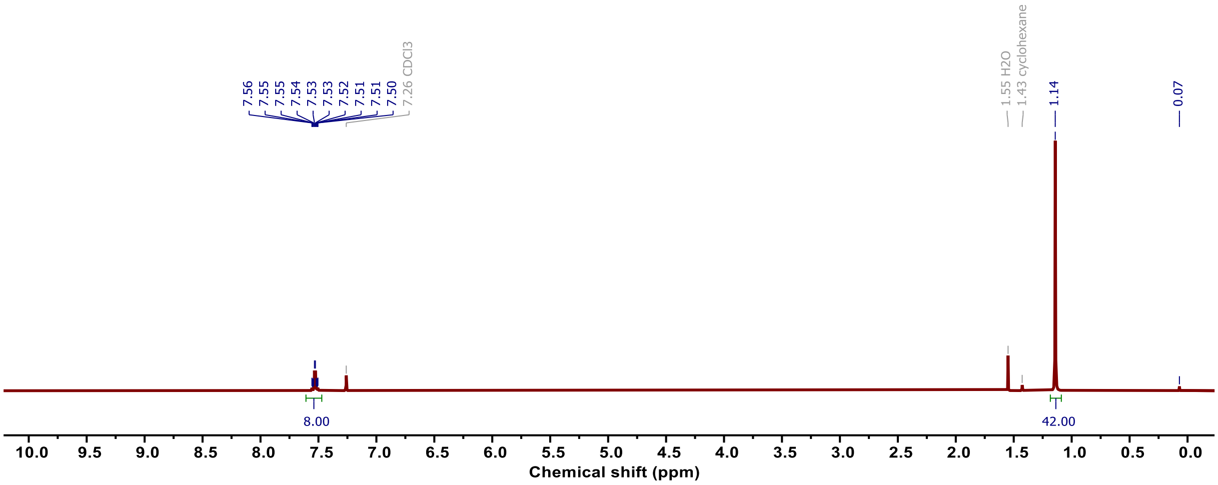


*Figure S 30: $^{1}$H NMR spectrum of bis(triisopropylsilylacetylene)-biphenyl in $CDCl_3$.*

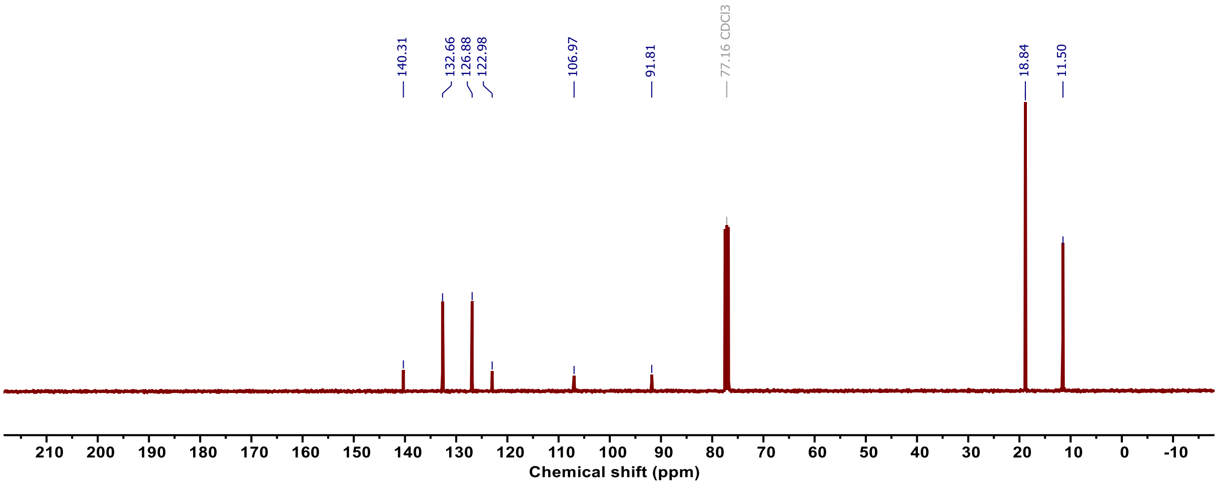


*Figure S 31: $^{13}$C NMR spectrum of bis(triisopropylsilylacetylene)-biphenyl in $CDCl_3$.*

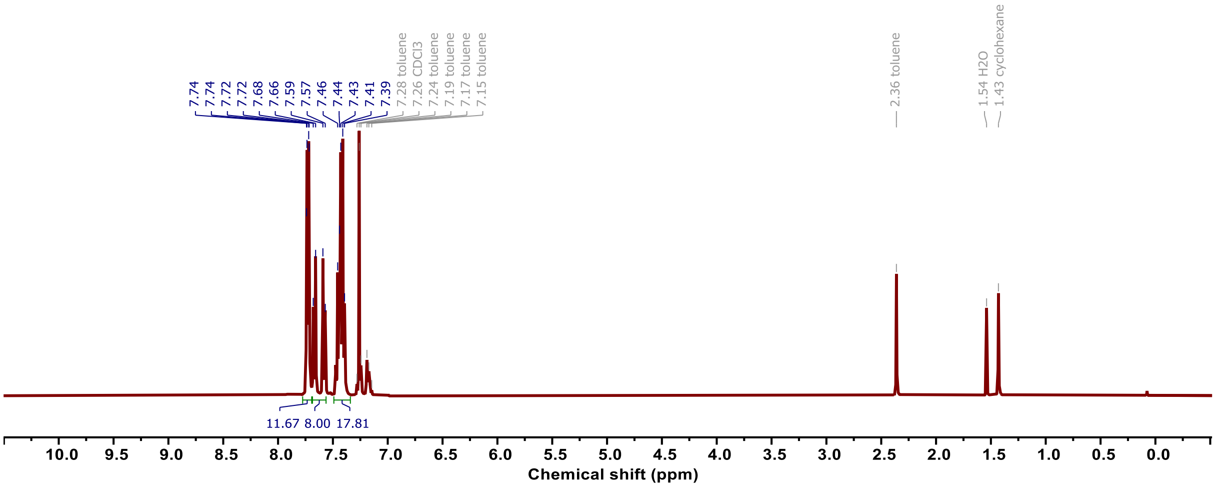


*Figure S 32: $^{1}$H NMR spectrum of bis(triphenylsilylacetylene)-biphenyl in $CDCl_3$. The signals in the 7.28– 7.15 ppm region are due to the aromatic protons of co-crystallized toluene.*

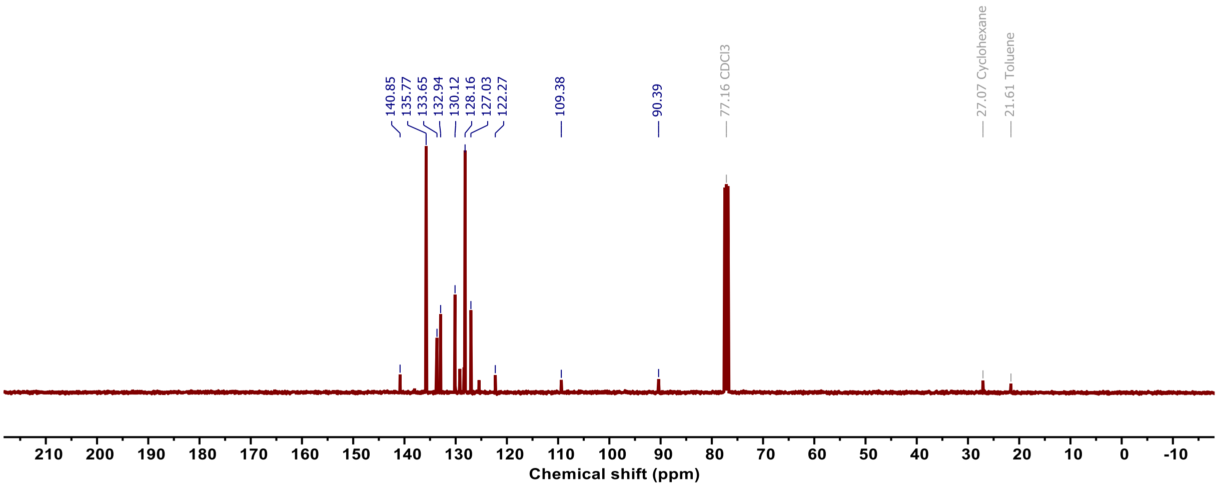


*Figure S 33: $^{13}$C NMR spectrum of bis(triphenylsilylacetylene)-biphenyl in $CDCl_3$.*

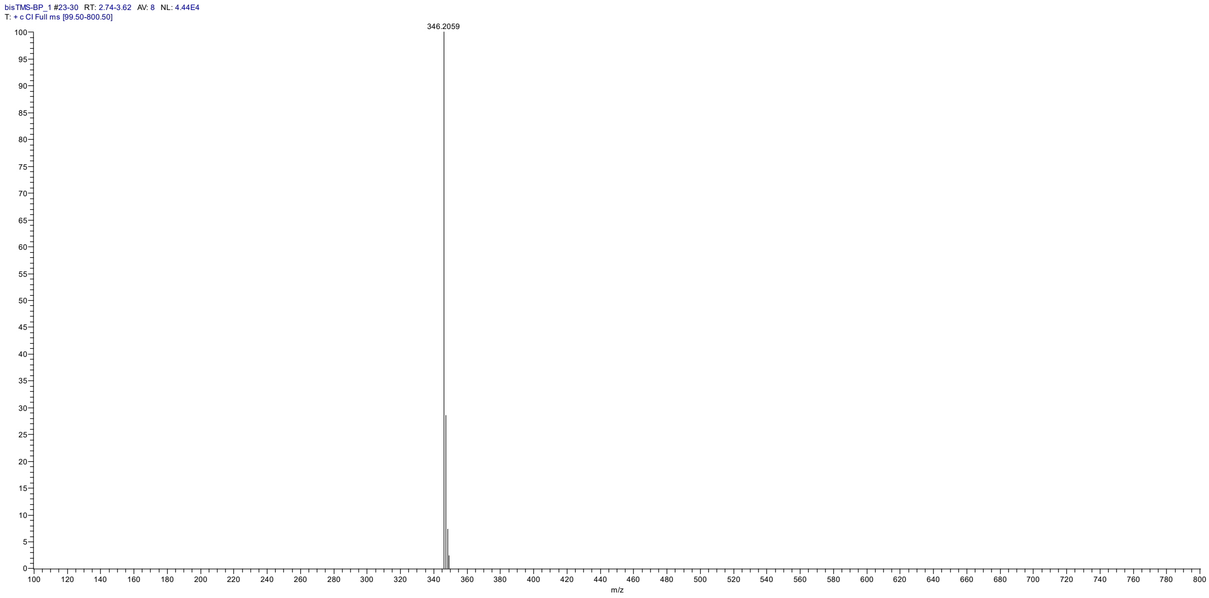


*Figure S 34: FD-MS spectrum of bis(trimethylsilylacetylene)-biphenyl.*

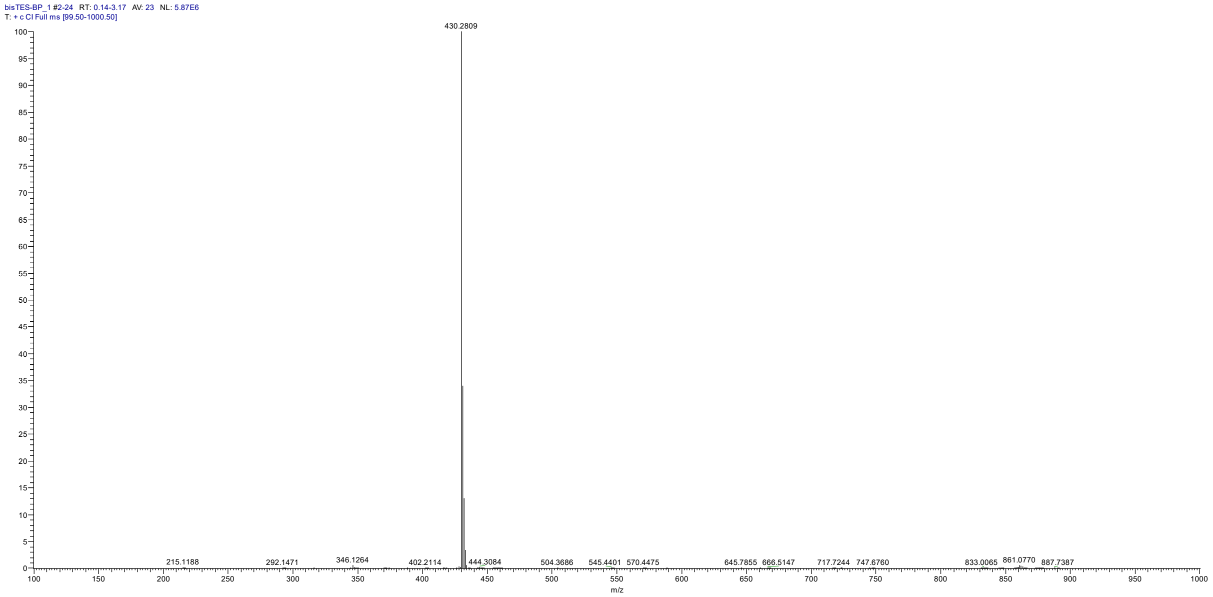


*Figure S 35: FD-MS spectrum of bis(triethylsilylacetylene)-biphenyl.*

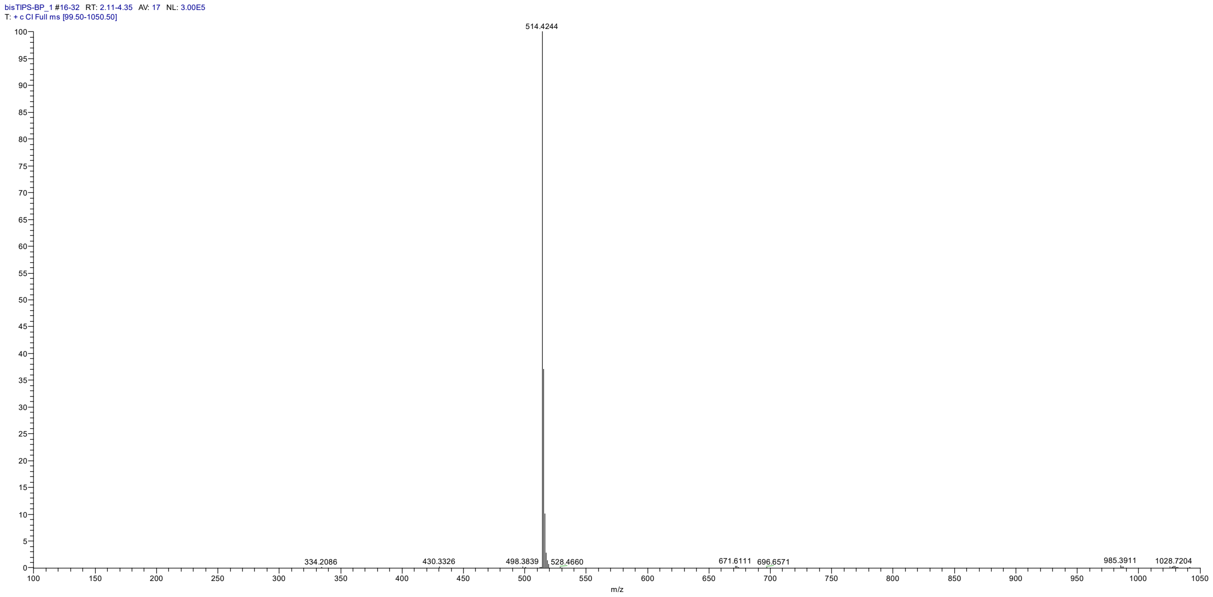


*Figure S 36: FD-MS spectrum of bis(triisopropylsilylacetylene)-biphenyl.*

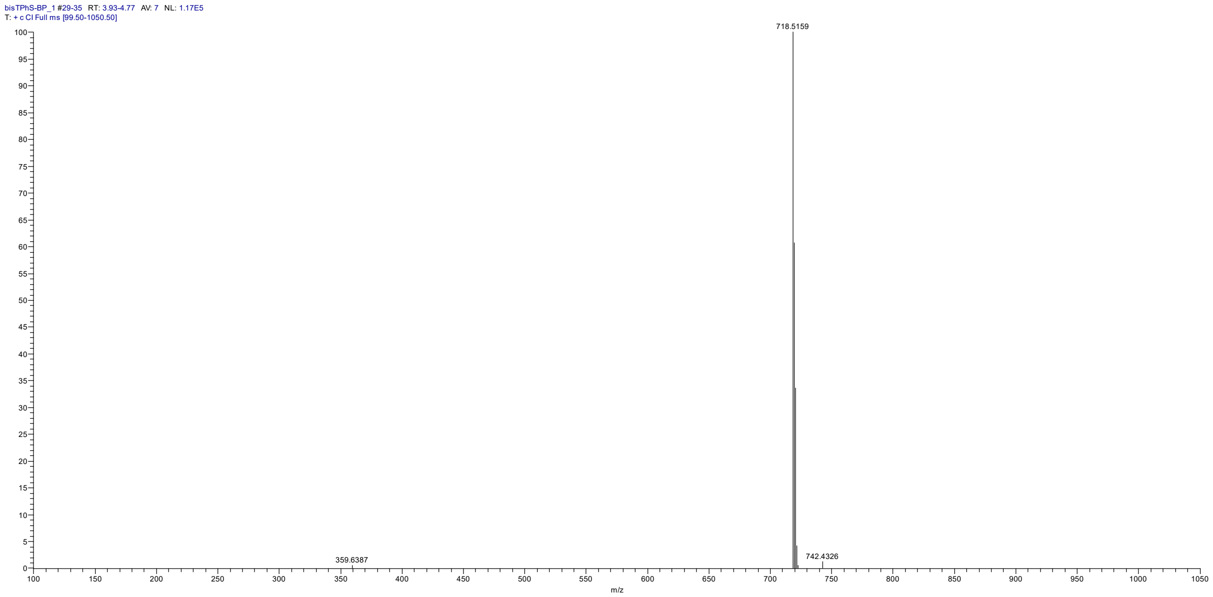


*Figure S 37: FD-MS spectrum of bis(triphenylsilylacetylene)-biphenyl.*

## 5. X-ray crystallography

The single X-ray data for (bTPhS-BP) were collected with STOE STADIVARI diffractometer using an AXO Mo micro-focus source with Mo–Kα radiation (λ = 0.71073 Å) equipped with DECTRIS EIGER2 1M CdTe detector. Data collection was performed at 120(2) K using an OXFORD Cryostream 700. Data integration, reduction and absorption correction was done with the STOE X- Area/LANA software package. The structure was solved with SHELXT and refined by the full-matrix method based on $F^2$ using SHELXL implemented in the OLEX2 graphical interface.[25–27] All non-hydrogen atoms were refined anisotropically, while the hydrogen atoms were fixed geometrically at calculated distances and allowed to ride on the parent atoms. Final crystallographic data and values of $R_1$ and $wR_2$ are listed in Table S 5. CCDC 2464155 contains the supplementary crystallographic data for this paper. This data can be obtained free of charge from The Cambridge Crystallographic Data Centre.

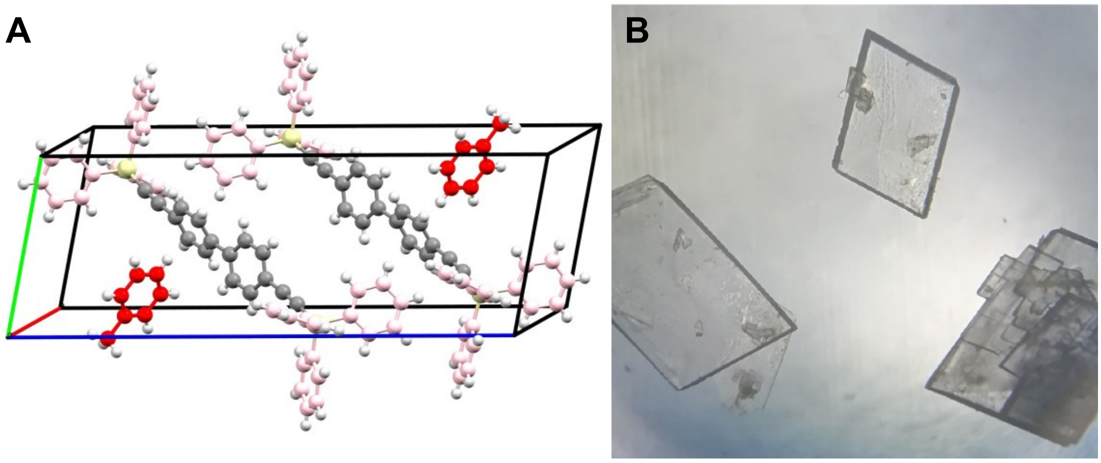


*Figure S 38: **A** unit cell of a bis(triphenylsilylacetylene)-biphenyl/toluene co-crystallization. **B**: Photograph of a bis(triphenylsilylacetylene)-biphenyl/toluene co-crystal through a microscope.*

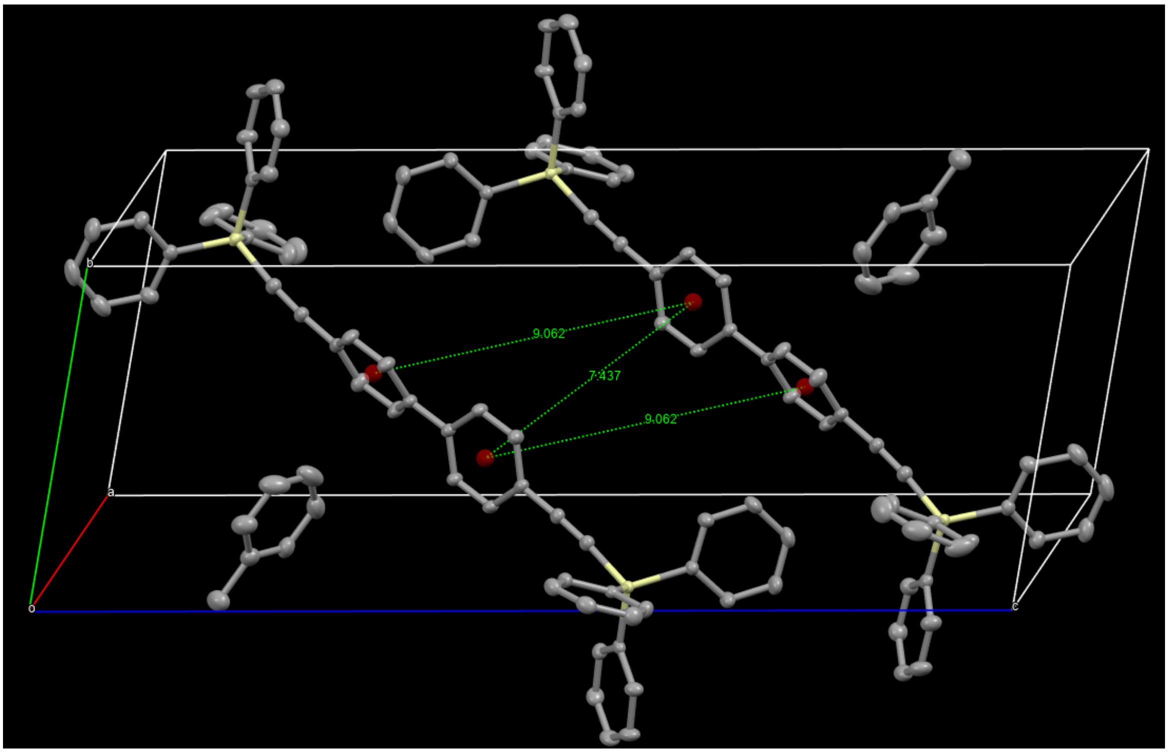


*Figure S 39: Unit cell of a bis(triphenylsilylacetylene)-biphenyl/toluene co-crystallization. The median distances from the center of one aromatic core unit to the aromatic core of the next unit of the aromatic systems are marked in green.*

*Table S 5: Crystal data and structure refinement for bis(triphenylsilylacetylene)-biphenyl/toluene co-crystal.*

| **Complex** | **1 · $C_7H_8$** |
|---|---|
| Formula | C59H46Si2 |
| Formula weight / gmol$^{-1}$ | 811.14 |
| Color & Habit | colorless plate |
| Crystal dimension / mm | 0.07 x 0.45 x 0.87 |
| Temp / K | 120(2) |
| Crystal system | triclinic |
| Space group | *P*-1 |
| *a* / Å | 8.8195(4) |
| *b* / Å | 9.8131(4) |
| *c* / Å | 27.3013(11) |
| *α* ° | 79.617(3) |
| *β* ° | 80.530(3) |
| *γ* ° | 75.462(3) |
| *V* / Å$^3$ | 2232.00(17) |
| *Z* | 2 |
| *P*calc / g cm$^{-3}$) | 1.207 |
| *μ* / mm$^{-1}$ | 0.119 |
| *F*(000) | 856 |
| no. of rflns collected | 44713 |
| no. of unique rflns | 13355 |
| no. rflns ($I > 2\sigma(I)$) | 11043 |
| $R_{int}$ | 0.0544 |
| no. params | 551 |
| restrains | 0 |
| GOOF | 1.051 |
| $R_1^a$, $wR_2^b$ ($I > 2\sigma(I)$) | 0.0663, 0.1813 |

| | |
|---|---|
| $R_1{}^a$, $wR_2{}^b$ (all data) | 0.0765, 0.1947 |
| CCDC | 2464155 |

$^aR1 = \Sigma||Fo| - |Fc||/\Sigma|Fo|$. $^bwR_2 = [\Sigma w(F_o{}^2 - F_c{}^2)^2/\Sigma w(F_o{}^2)^2]^{1/2}$.

## 6. References


(1) Zähringer, T. J. B.; Bertrams, M.-S.; Kerzig, C. Purely Organic Vis-to-UV Upconversion with an Excited Annihilator Singlet beyond 4 eV. *J. Mater. Chem. C* **2022**, *10* (12), 4568–4573. https://doi.org/10.1039/D1TC04782E.

(2) Gottlieb, H. E.; Kotlyar, V.; Nudelman, A. NMR Chemical Shifts of Common Laboratory Solvents as Trace Impurities. *J. Org. Chem.* **1997**, *62* (21), 7512–7515. https://doi.org/10.1021/jo971176v.

(3) Fulmer, G. R.; Miller, A. J. M.; Sherden, N. H.; Gottlieb, H. E.; Nudelman, A.; Stoltz, B. M.; Bercaw, J. E.; Goldberg, K. I. NMR Chemical Shifts of Trace Impurities: Common Laboratory Solvents, Organics, and Gases in Deuterated Solvents Relevant to the Organometallic Chemist. *Organometallics* **2010**, *29* (9), 2176–2179. https://doi.org/10.1021/om100106e.

(4) Singh-Rachford, T. N.; Castellano, F. N. Photon Upconversion Based on Sensitized Triplet–Triplet Annihilation. *Coord. Chem. Rev.* **2010**, *254* (21–22), 2560–2573. https://doi.org/10.1016/j.ccr.2010.01.003.

(5) Yanai, N.; Suzuki, K.; Ogawa, T.; Sasaki, Y.; Harada, N.; Kimizuka, N. Absolute Method to Certify Quantum Yields of Photon Upconversion via Triplet–Triplet Annihilation. *J. Phys. Chem. A* **2019**, *123* (46), 10197–10203. https://doi.org/10.1021/acs.jpca.9b08636.

(6) Murakami, Y.; Kamada, K. Kinetics of Photon Upconversion by Triplet–Triplet Annihilation: A Comprehensive Tutorial. *Phys. Chem. Chem. Phys.* **2021**, *23* (34), 18268–18282. https://doi.org/10.1039/D1CP02654B.

(7) Oskouei, A. A.; Bräm, O.; Cannizzo, A.; Van Mourik, F.; Tortschanoff, A.; Chergui, M. Ultrafast UV Photon Echo Peak Shift and Fluorescence up Conversion Studies of Non-Polar Solvation Dynamics. *Chem. Phys.* **2008**, *350* (1–3), 104–110. https://doi.org/10.1016/j.chemphys.2008.01.029.

(8) Pope, M.; Swenberg, C. E.; Pope, M. *Electronic Processes in Organic Crystals and Polymers*, 2. ed.; Monographs on the physics and chemistry of materials; Oxford University Press: New York, 1999.

(9) Edhborg, F.; Bildirir, H.; Bharmoria, P.; Moth-Poulsen, K.; Albinsson, B. Intramolecular Triplet–Triplet Annihilation Photon Upconversion in Diffusionally Restricted Anthracene Polymer. *J. Phys. Chem. B* **2021**, *125* (23), 6255–6263. https://doi.org/10.1021/acs.jpcb.1c02856.

(10) Olesund, A.; Gray, V.; Mårtensson, J.; Albinsson, B. Diphenylanthracene Dimers for Triplet–Triplet Annihilation Photon Upconversion: Mechanistic Insights for Intramolecular Pathways and the Importance of Molecular Geometry. *J. Am. Chem. Soc.* **2021**, *143* (15), 5745–5754. https://doi.org/10.1021/jacs.1c00331.

(11) Sun, W.; Ronchi, A.; Zhao, T.; Han, J.; Monguzzi, A.; Duan, P. Highly Efficient Photon Upconversion Based on Triplet–Triplet Annihilation from Bichromophoric Annihilators. *J. Mater. Chem. C* **2021**, *9* (40), 14201–14208. https://doi.org/10.1039/D1TC01569A.

(12) Ji, H.; Luo, Z.; Yang, X.; Jin, X.; Zhao, T.; Duan, P. Chiral Dual-Annihilator Model for Controllable Photon Upconversion and Multi-Dimensional Optical Modulation. *Nat. Commun.* **2025**, *16* (1), 4952. https://doi.org/10.1038/s41467-025-60290-7.

(13) Radiunas, E.; Naimovičius, L.; Baronas, P.; Jozeliūnaitė, A.; Orentas, E.; Kazlauskas, K. CN-Tuning: A Pathway to Suppress Singlet Fission and Amplify Triplet-Triplet

Annihilation Upconversion in Rubrene. *Adv. Opt. Mater.* **2025**, *13* (12), 2403032. https://doi.org/10.1002/adom.202403032.
(14) Rigsby, E. M.; Miyashita, T.; Fishman, D. A.; Roberts, S. T.; Tang, M. L. CdSe Nanocrystal Sensitized Photon Upconverting Film. *RSC Adv.* **2021**, *11* (49), 31042–31046. https://doi.org/10.1039/D1RA06562A.
(15) Neese, F. The ORCA Program System. *WIREs Comput. Mol. Sci.* **2012**, *2* (1), 73–78. https://doi.org/10.1002/wcms.81.
(16) Hanwell, M. D.; Curtis, D. E.; Lonie, D. C.; Vandermeersch, T.; Zurek, E.; Hutchison, G. R. Avogadro: An Advanced Semantic Chemical Editor, Visualization, and Analysis Platform. *J. Cheminformatics* **2012**, *4* (1), 17. https://doi.org/10.1186/1758-2946-4-17.
(17) Rajasekar, M. Recent Development in Fluorescein Derivatives. *J. Mol. Struct.* **2021**, *1224*, 129085. https://doi.org/10.1016/j.molstruc.2020.129085.
(18) Kolthoff, I. M.; Chantooni, M. K.; Smagowski, Henryk. Acid-Base Strength in N,N-Dimethylformamide. *Anal. Chem.* **1970**, *42* (13), 1622–1628. https://doi.org/10.1021/ac60295a013.
(19) Hushvaktov, H.; Jumabaev, A.; Doroshenko, I.; Absanov, A. Raman Spectra and Non-Empirical Calculations of Dimethylformamide Molecular Clusters Structure. *Vib. Spectrosc.* **2021**, *117*, 103315. https://doi.org/10.1016/j.vibspec.2021.103315.
(20) Zhou, Q.; Wirtz, B. M.; Schloemer, T. H.; Burroughs, M. C.; Hu, M.; Narayanan, P.; Lyu, J.; Gallegos, A. O.; Layton, C.; Mai, D. J.; Congreve, D. N. Spatially Controlled UV Light Generation at Depth Using Upconversion Micelles. *Adv. Mater.* **2023**, *35* (46), 2301563. https://doi.org/10.1002/adma.202301563.
(21) Peng, Y.; Li, J.-Y.; Qi, F.; Guo, D.-X.; Li, Y.-Z.; Feng, H.-J.; Jiang, L.-H.; Zhang, M.-Y.; Liu, Y.-X.; Zeng, L.; Huang, L. Highly Effective Near-Infrared to Blue Triplet–Triplet Annihilation Upconversion Nanoparticles for Reversible Photobiocatalysis. *Nano Lett.* **2025**, *25* (13), 5291–5298. https://doi.org/10.1021/acs.nanolett.5c00117.
(22) Olesund, A.; Ghasemi, S.; Moth-Poulsen, K.; Albinsson, B. Bulky Substituents Promote Triplet–Triplet Annihilation Over Triplet Excimer Formation in Naphthalene Derivatives. *J. Am. Chem. Soc.* **2023**, *145* (40), 22168–22175. https://doi.org/10.1021/jacs.3c08115.
(23) Monguzzi, A.; Tubino, R.; Hoseinkhani, S.; Campione, M.; Meinardi, F. Low Power, Non-Coherent Sensitized Photon up-Conversion: Modelling and Perspectives. *Phys. Chem. Chem. Phys.* **2012**, *14* (13), 4322. https://doi.org/10.1039/c2cp23900k.
(24) Edhborg, F.; Olesund, A.; Albinsson, B. Best Practice in Determining Key Photophysical Parameters in Triplet–Triplet Annihilation Photon Upconversion. *Photochem. Photobiol. Sci.* **2022**, *21* (7), 1143–1158. https://doi.org/10.1007/s43630-022-00219-x.
(25) Sheldrick, G. M. *SHELXT* – Integrated Space-Group and Crystal-Structure Determination. *Acta Crystallogr. Sect. Found. Adv.* **2015**, *71* (1), 3–8. https://doi.org/10.1107/S2053273314026370.
(26) Sheldrick, G. M. Crystal Structure Refinement with *SHELXL*. *Acta Crystallogr. Sect. C Struct. Chem.* **2015**, *71* (1), 3–8. https://doi.org/10.1107/S2053229614024218.
(27) Dolomanov, O. V.; Bourhis, L. J.; Gildea, R. J.; Howard, J. A. K.; Puschmann, H. *OLEX2* : A Complete Structure Solution, Refinement and Analysis Program. *J. Appl. Crystallogr.* **2009**, *42* (2), 339–341. https://doi.org/10.1107/S0021889808042726.
